# Correlation-driven attosecond photoemission delay in the plasmonic excitation of C_60_ fullerene

**DOI:** 10.1126/sciadv.ads0494

**Published:** 2025-02-12

**Authors:** Shubhadeep Biswas, Andrea Trabattoni, Philipp Rupp, Maia Magrakvelidze, Mohamed El-Amine Madjet, Umberto De Giovannini, Mattea C. Castrovilli, Mara Galli, Qingcao Liu, Erik P. Månsson, Johannes Schötz, Vincent Wanie, Pawel Wnuk, Lorenzo Colaizzi, Daniele Mocci, Maurizio Reduzzi, Matteo Lucchini, Mauro Nisoli, Angel Rubio, Himadri S. Chakraborty, Matthias F. Kling, Francesca Calegari

**Affiliations:** ^1^ Max Planck Institute of Quantum Optics, Hans-Kopfermann-Straße 1, D-85748 Garching, Germany.; ^2^ Department of Physics, Ludwig-Maximilians-Universität München, Am Coulombwall 1, D-85748 Garching, Germany.; ^3^ SLAC National Accelerator Laboratory, 2575 Sand Hill Rd., Menlo Park, CA 94025, USA.; ^4^ Center for Free-Electron Laser Science CFEL, Deutsches Elektronen-Synchrotron DESY, Notkestrasse 85, 22607 Hamburg, Germany.; ^5^ Institute of Quantum Optics, Leibniz Universität Hannover, Welfengarten 1, 30167 Hannover, Germany.; ^6^ Department of Physics, Villanova University, Villanova, PA 19085, USA.; ^7^ Department of Physics, Temple University, Philadelphia, PA 19122, USA.; ^8^ Department of Natural Sciences, D.L. Hubbard Center for Innovation, Northwest Missouri State University, Maryville, MO 64468, USA.; ^9^ Bremen Center for Computational Materials Science, University of Bremen, Bremen, Germany.; ^10^ Max Planck Institute for the Structure and Dynamics of Matter, Luruper Chaussee 149, D-22761 Hamburg, Germany.; ^11^ Università degli Studi di Palermo, Via Archirafi 36, 90123 Palermo, Italy.; ^12^ Institute for Photonics and Nanotechnologies CNR-IFN, P.za Leonardo da Vinci 32, 20133 Milano, Italy.; ^13^ Istituto Struttura della Materia, ISM-CNR, Monterotondo Scalo, 00016 Roma, Italy.; ^14^ Department of Physics, Politecnico di Milano, Piazza Leonardo da Vinci 32, 20133 Milano, Italy.; ^15^ Center for Computational Quantum Physics (CCQ), The Flatiron Institute, New York, NY, USA.; ^16^ Applied Physics Department, Stanford University, 348 Via Pueblo, Stanford, CA 94305, USA.; ^17^ The Hamburg Centre for Ultrafast Imaging, Universität Hamburg, 149 Luruper Chaussee, 22761 Hamburg, Germany.; ^18^ Physics Department, University of Hamburg, Luruper Chaussee 149, 22761 Hamburg, Germany.

## Abstract

Extreme light confinement in plasmonic nanosystems enables novel applications in photonics, sensor technology, energy harvesting, biology, and quantum information processing. Fullerenes represent an extreme case for nanoplasmonics: They are subnanometer carbon-based molecules showing high-energy and ultrabroad plasmon resonances; however, the fundamental mechanisms driving the plasmonic response and the corresponding collective electron dynamics are still elusive. Here, we uncover the dominant role of electron correlations in the dynamics of the giant plasmon resonance (GPR) of the subnanometer system C_60_ by using attosecond photoemission chronoscopy. We find a characteristic photoemission delay of up to about 300 attoseconds that is purely induced by coherent large-scale electron correlations in the plasmonic potential. These results provide insights into the nature of the plasmon resonances in subnanometer systems and open perspectives for advancing nanoplasmonic applications.

## Teaser

Attosecond photoemission chronoscopy reveals the role of electron correlations in the plasmonic dynamics of C_60_ fullerene.

## INTRODUCTION

Plasmonics offers a way to achieve extreme light confinement and control surpassing the constraint of the diffraction limit. It harnesses the ability to confine light within the nanoscale size by hybrid “quasiparticle” states involving light and collective electron excitation. In this context, the studies of surface plasmons and the even more peculiar giant plasmon resonances (GPRs) have already enabled transformational applications including solar energy harvesting, ultrafine sensor technology, and controlled photocatalysis (*1*–*3*).

**Fig. 1.**
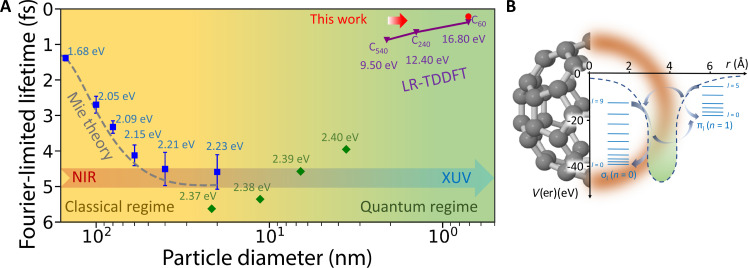
Plasmonic response and electron correlation induced GPR in C_60_. (**A**) Fourier-limited lifetime (FLL) of plasmonic resonances as a function of particle diameter. The blue (*4*) and green (*34*) points represent experimental values obtained from literature for nanosystems of different sizes. The grey dashed curve represents the Mie theory prediction (*4*) applicable mostly in the classical region. All these data are obtained from the linewidth measurements which were converted to FLL by the relation $\text{FLL}=\frac{2\hbar }{\Gamma }$, where ℏ is reduced Planck’s constant and Γ is the plasmon resonance linewidth. The purple points represent the linear-response time-dependent density functional theory (LR-TDDFT) predicted FLL for nano or sub-nano fullerenes, which reside in the quantum regime (*35*). The present statistically averaged LR-TDDFT time delay is shown as the red full circle. The values written adjacent to the symbols represent plasmon peak positions in the spectral domain from which it is evident that the plasmon central frequency can be scaled from NIR to XUV regions with the nanoparticle size. (**B**) Spherical shell-like distribution of delocalized electrons around C_60_ exhibits a collective giant plasmon excitation at around 20 eV. The jellium-based DFT potential depicted as a function of radial coordinate of C_60_ provides the energetics of the involved quantum states, which can be classified into π and σ characters. The configuration interactions (schematically represented by the arrows) among the electrons occupying these states give rise to the GPR.

The central energy of the plasmon resonance and its linewidth are relevant parameters because they determine, for instance, the frequency and lifetime of the light confinement. Usually, these parameters scale with the size, shape, and composition of the plasmonic nanosystem. As an example, in Fig. 1A, we report the case of gold nanospheres: The plasmon central energy systematically evolves from near-infrared (NIR) to the visible spectral region with increasing particle diameter, while the Fourier-limited lifetime (FLL)—simply expressed in Fig. 1A as the Fourier transformation of the corresponding linewidth—moves toward longer values. This is true only for particles with diameter larger than ~10 nm, and the trend can be well characterized by classical Mie-type models involving electromagnetic theory and Drude-like dielectric response (*4*–*6*). For smaller particles, i.e., approaching the nanometer or subnanometer, the above-mentioned classical interpretation breaks down (*3*), and the plasmonic response in this case is poorly understood (*5*–*7*). In the pioneering work by Scholl *et al.* (*5*), it was explicitly shown that the peak energy of the resonance for few-nanometer particles deviates from the Mie theory prediction. It was also proposed that the incorporation of an ensemble of incoherent quantum Lorentzian-type (single electron excitation) oscillators could explain the discrepancy, and the linewidth of the resonances was reproduced by a fitting procedure involving size-dependent damping. Although some of the aspects of the plasmonic resonance (including the peak energy) were discussed for ultra-small nanoparticles (*5*, *6*, *8*), the precise origin and evolution of the linewidth was not scrutinized in detail. Among the family of subnanometer plasmonic particles, fullerenes represent an extreme case (see the top right corner of Fig. 1A). They are cage-shaped single molecules of carbon atoms featuring a number of peculiar properties: (i) They show giant plasmonic resonances in the continuum at extreme-ultraviolet energies (read below for a precise definition of plasmons in fullerenes), thus triggering photoemission (*9*–*11*); (ii) the linewidth of these resonances is ultrabroadband, suggesting possible attosecond lifetimes subjected to coherent superposition. The ultrafast study of the plasmonic response of these systems, still mostly unexplored, represents an excellent platform to understand the fundamental physical mechanisms of collective electron motion in subnanometer particles. Furthermore, it sets an extreme condition for nanoplasmonics, for which the definition itself of plasmon is still debated and requires a deep understanding of the underlying electron dynamics.

To this purpose, direct time-resolved measurements of the plasmonic dynamics with attosecond resolution are then required. Inferring the plasmon lifetime from the corresponding linewidth, i.e., from frequency-domain measurements, can be misleading especially for ultrabroadband resonances. The possible coherent superposition of narrowband peaks in the total resonant cross section, or simply the phase of the resonance, makes the direct linewidth-to-lifetime assumption inconsistent.

In our work, we experimentally and theoretically studied the plasmon dynamics for the most abundant fullerene C_60_ (*12*). In particular, we measured the photoemission delay following the photoexcitation of the GPR by an extreme ultraviolet (XUV) attosecond pulse. We found that the electron propagating during photoionization in the large-scale correlation-induced plasmonic potential accumulates a photoemission delay ranging from a minimum of 50 as to about 300 as over a vast kinetic energy range. This delay arises from coherent large-scale electron correlations. This observation demonstrates that electron correlations play a relevant role in the plasmon response of C_60_ and suggests that plasmonics at the subnanometer level should be understood beyond the classical picture of collective single-particle electron motion.

## RESULTS

Among the various fullerenes, the most common form is C_60_, and it consists of a nearly delocalized cloud of 240 valence electrons around its carbon skeleton (see Fig. 1B). For such an ultra-small system, the relevant quantum mechanical energy levels are discretized (*7*, *13*, *14*). Furthermore, the coherent dynamics among its π and σ band states (Fig. 1B) resulting from photoexcitation gives rise to a collective electron motion that has been identified in literature as a GPR (*15*). This resonance has a high excitation energy of about 20 eV, well above the ionization threshold (7.6 eV), and a bandwidth exceeding 10 eV (*15*, *16*). The many-body interaction underlying this resonance includes two main components: (i) multiple, incoherent single-electron excitation channels with narrow linewidths and therefore longer lifetimes, and (ii) a coherent, correlation-driven, collective excitation channel with ultrabroad bandwidth resulting in an ultrashort lifetime (*14*). Therefore, an ultrafast measurement can capture the later effect relatively uncontaminated. Theoretically, classical models mimicking the collective electron motion around the C_60_ cage have been able to reproduce the excitation energy of the resonance with small deviation, but not its ultrabroad bandwidth (*17*). Quantum theories, instead, while better matching the experimentally observed resonance shape, intrinsically imply the interplay between the incoherent and the coherent dynamics mentioned above (*13*, *14*). Experimentally, the GPR of C_60_ has been studied by differential absorption or ionization cross section measurements and with angle-resolved photoelectron spectroscopy (*9*, *16*, *18*). While these studies provided knowledge about the spectral features of the resonance, they did not allow the slow incoherent component to be disentangled from fast correlation-driven coherent dynamics (*14*). As a result, the electronic character of the GPR in C_60_ and, particularly, the role of correlations in the plasmonic response are still an open question.

The photoionization of GPR in C_60_ can be viewed to occur from the coupling between the collective excitation, embedded in the ionization continuum, with the simultaneous and degenerate ionization channels. Therefore, the plasmonic response of the molecule is imprinted in the time spent by the electrons to reach the continuum. The strong fingerprint of the C_60_ plasmon dynamics and decay in attosecond photoemission delays was suggested earlier (*19*). In the framework of quantum mechanics, photoemission can intuitively be understood within half-scattering theory: During photoionization, the outgoing electron scatters on the surrounding static and time-dependent correlation-induced potential. Each effect induces a phase shift in the electron emission amplitude, all of which are additive (*20*). The derivative of the overall phase shift with respect to energy can be associated with a photoemission time delay, which is usually referred to as the Eisenbud-Wigner-Smith (EWS) delay (*21*–*23*). For the case of the GPR in C_60_, the excited plasmon is expected to reshape the potential experienced by the outgoing electron, therefore affecting the total EWS delay. In particular, the presence of electronic correlations, and their primary role in the GPR, can be demonstrated by measuring the EWS delay with attosecond precision (*23*–*25*).

**Fig. 2.**
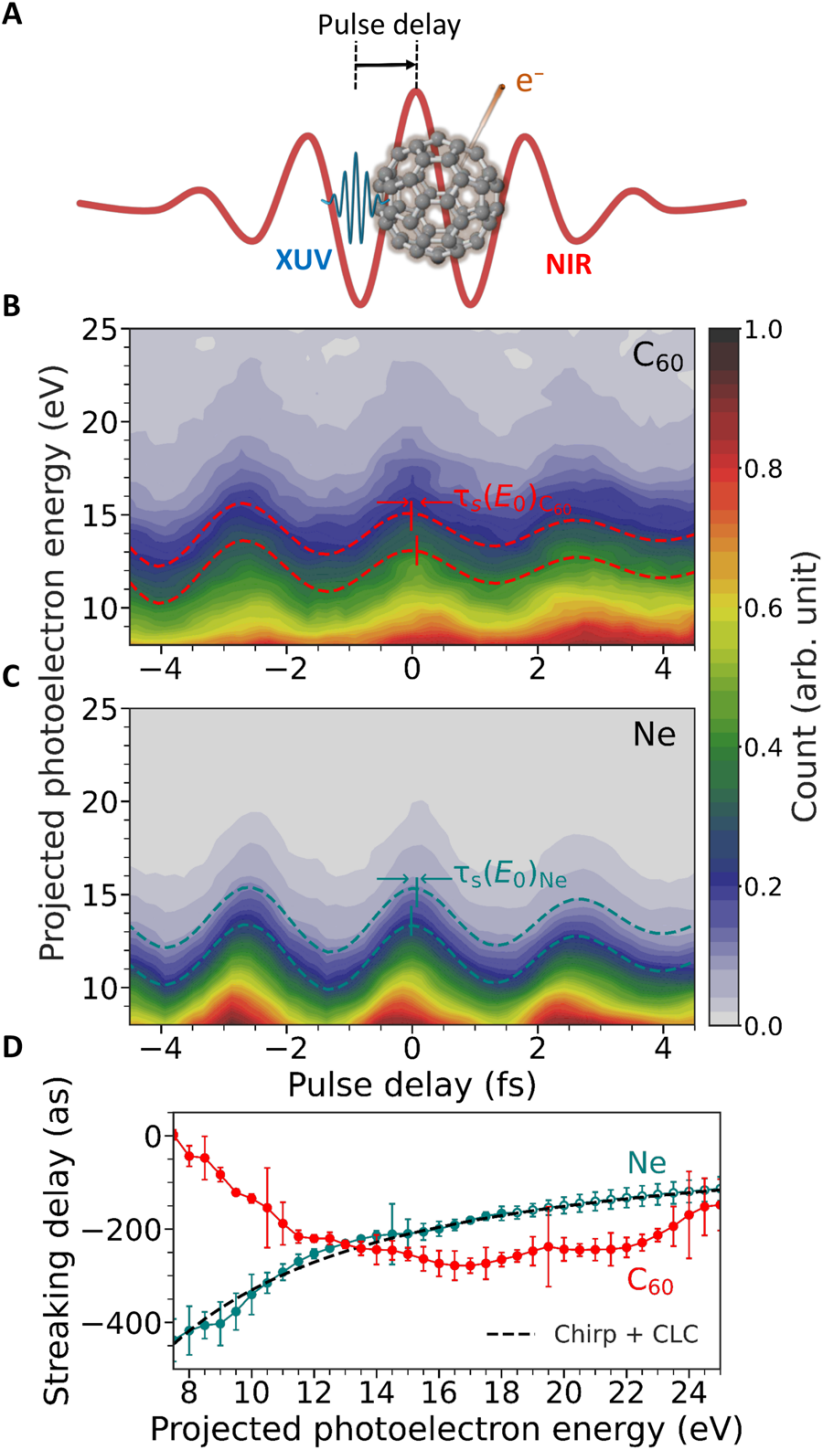
Attosecond photoemission delays. (**A**) Synchronized XUV and NIR pulses were used for ionizing the C_60_ around the GPR and clocking the emission timing, respectively. (**B** and **C**) Experimentally measured streaking traces for C_60_ and neon (2p), respectively. The representative streaking curves extracted at given projected photoelectron energies are shown, whose relative phase difference translates into the projected photoelectron energy–dependent streaking delays [τ_s_(*E*_0_)]. (**D**) Extracted streaking delays as function of projected photoelectron energy for C_60_ and neon (2p). These results are obtained by weighted averaging over the output of five independent measurements, where each measurement also contributes two separate datasets corresponding to two opposite directions along the laser polarization axis. The streaking delay curves share a common time zero reference, which, however, is unknown. The error bars represent the weighted SD including all the measurements. The open symbols for the neon results in the higher photoelectron energy side (after 18 eV) indicate the values extracted through extrapolation (see the Supplementary Materials for details). The chirp + CLC contribution for neon is shown as reference.

To measure the photoemission delay in C_60_, we used attosecond streaking metrology (*24*, *26*, *27*), which is schematically illustrated in Fig. 2A and described in detail in the Supplementary Materials. In brief, a sub–300-attosecond XUV pump pulse was interferometrically combined with a carrier-envelope phase (CEP) stabilized sub–5-femtoseconds NIR probe pulse (*27*). The spectrum of the generated XUV pulse (covering from 15 to 35 eV) overlapped with most of the GPR. The two synchronized pulses at variable pulse delay were then focused onto a gas-phase target of isolated C_60_ molecules.

The C_60_ molecules were photoionized by the attosecond XUV pulse in the single-photon regime, and a delay-dependent phase was imprinted in the photoelectron momentum by the NIR field in an implementation of attosecond streaking spectroscopy (*24*, *27*). The NIR peak intensity was set below 5 × 10^12^ W/cm^2^ to avoid any spurious contribution of above-threshold ionization electrons in the energy region of interest of our experiment. The emitted photoelectrons were collected in a velocity map imaging (VMI) spectrometer as a function of the delay between XUV pump and NIR probe (*27*). By integrating the acquired 2D projected photoelectron momentum distributions over a small angle along the laser polarization axis, we obtain the projected photoelectron kinetic energy distributions for each delay step, resulting in the C_60_ streaking spectrogram shown in Fig. 2B. A second spectrogram (c.f. Fig. 2C) was acquired for neon in the same way and used as relative timing reference. The acquisition time of the streaking trace for C_60_ was 10 times longer than for Ne due to the inherently low target density of the fullerene sample. Aside from the effect of the NIR vector potential giving rise to the overall shape of the spectrogram, energy-dependent temporal shifts are known to be induced by the EWS delay (*23*, *24*, *28*), the effect of the Coulomb laser coupling (CLC) (*29*), and the chirp of the XUV attosecond pulse (*27*). For C_60_, additionally, a supplementary phase is induced by the GPR itself and another by the presence of a NIR-induced dipolar near-field. Regarding the phase induced by the GPR, the contribution of long-lived electronic channels should emerge as sharp, nonstreaked, photoelectron lines in the spectrogram of Fig. 2B before the interaction with IR vector potential in this case would occur later than the measured delay window. We did not observe these features in our experiment, suggesting that the broad, coherent, excitation channel plays a major role in the GPR dynamics. The overall temporal phase, here defined as the streaking delay (τ_s_), can be extracted for C_60_ and neon directly from the experimental spectrograms by fitting isocontour lines for different photoelectron energies. In Fig. 2 (B and C), representative isocontour lines are shown at two different photoelectron energies, indicating their relative τ_s_. In the Supplementary Materials, further details are provided about the synchronization of the two targets and the isoline analysis.

The streaking delays as a function of photoelectron energy for the two targets are shown in Fig. 2D. The two curves display opposite trends, with the one of neon (*28*, *29*) showing a typical trend determined by the chirp of the attosecond pulse, the EWS delay on Ne and the CLC contribution. By performing dedicated calculations of the photoemission delay in neon, a chirp of 12,500 as^2^ was estimated for the attosecond pulse. Moreover, we note that the extracted streaking delays are defined up to an arbitrary offset. Because of the synchronization between the measurements on C_60_ and neon (see the Supplementary Materials for details), however, this offset is identical for both targets.

**Fig. 3.**
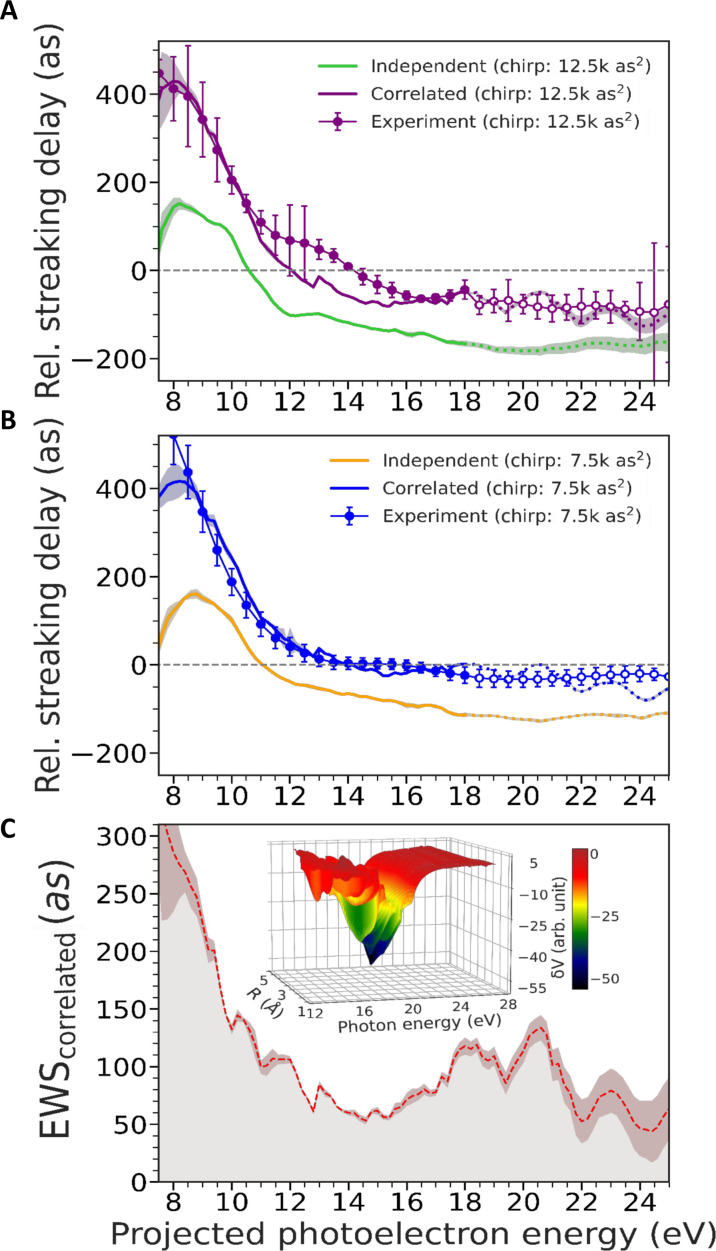
Relative streaking delay and effect of the GPR on photoemission delay. (**A** and **B**) Relative streaking delay for C_60_ in comparison to that for neon 2p emission for attochirp values of 12,000 as^2^ (A) and 7500 as^2^ (B). These data are obtained by weighted averaging over all the relative streaking delays extracted from each pair of C_60_ and neon measurements. The experimental results (markers) are compared with the simulations (solid curves), which comprise quantum mechanical DFT calculations and CTMC calculations. The CTMC simulations yield relative streaking delay contributions from XUV chirp, CLC and induced near field effects. The XUV response causing EWS delay is modeled by LR-TDDFT, which takes into account the collective excitation response. For comparison, the independent particle (mean-field) response is modeled by LR-DFT. To compare these with the experimental data in the relative streaking delay level, the CTMC results are added to the EWS results, yielding the purple and the green curves in (A) representing collective and independent responses, respectively. Similarly, the blue and yellow curves are displayed in (B) for the other attochirp. The error bars in the experimental curve represent the weighted SD (±σ) corresponding to all the above-mentioned measurements. The shaded regions in the theoretically simulated curves originate from the statistical ensemble of trajectories of the CTMC propagation. The open symbols for experimental data and the dotted line portions in the LR-DFT and LR-TDDFT results in the higher photoelectron energy side (after 18 eV) indicate the data extracted through extrapolation for neon (see the Supplementary Materials for details). (**C**) EWS delay contribution exclusively from the correlated excitation is extracted from the difference between the results of LR-TDDFT (correlated) and LR-DFT (mean-field) calculations. Also in this case, the CTMC propagation was included. The inset represents the induced potential due to GPR.

In the following discussion, we uncover the contribution of the large-scale electron correlations in the GPR of C_60_. For this, we consider relative streaking delays between C_60_ and neon (C_60_ − neon), which are free from any arbitrary delay offset. Because of the intrinsic synchronization between the measurements for both targets, the relative delay can be obtained with high accuracy and repeatability, facilitating the comparison of the experimental findings with theoretical predictions. The photoelectron energy dependence of the relative streaking delays is displayed in Fig. 3A, where theoretical predictions are also shown for comparison.

The theoretical simulations combine ab initio linear-response time-dependent density functional theory (LR-TDDFT) (*13*) with classical trajectory Monte-Carlo (CTMC) simulations (see the Supplementary Materials for further details). The LR-TDDFT theory is used to describe the time-dependent response of the electron density to the incident resonant oscillating field in a linear-response frame. The frequency-dependent change in the electron density induces a complex potential that accounts for electron correlations. For a given value of the photoelectron energy, the total photoemission delay is determined by averaging the delays weighted by the corresponding subshell cross section fractions. Our LR-TDDFT scheme with LB94 exchange-correlation functional describes the ionization continuum fairly accurately by producing the appropriate asymptotic behavior of the continuum wave function. In the framework of LR-TDDFT, the plasmon resonance is a broad line of pure many-body (collective) dynamics. The broad coherent part of the LR-TDDFT result is then translated into streaking delays by classical propagation simulations. The propagation part of the simulations, following earlier work (*27*), particularly incorporates the chirp of the attosecond pulse and the CLC to the streaking delay.

In addition, we have used a simple model based on classical electromagnetic theory for an intuitive understanding of the role of the fullerene polarizability (*17*). The high polarizability of C_60_ results in a coherent oscillation of the electron cloud upon interaction with the NIR field and, consequently, an oscillating near field with an asymptotic dipolar behavior. This near-field has an impact on the final phase of the propagating electron that was fully accounted for in the CTMC simulations. We note that similar results for the near-field induced streaking delay are obtained from the near-field constructed with both classical (*17*) and quantum density functional theory (DFT) (see figs. S3 and S5) (*30*).

The energy-dependent relative streaking delays obtained from combined LR-TDDFT and CTMC simulations [see Fig. 3A (purple curve)] were extracted in the same way as for the experiment. The experimental relative streaking delays are in remarkable agreement with the simulated curve. On the basis of the quantitative representation of the experimental data with the simulations, we also compared the data to simulations performed within linear-response DFT (LR-DFT) (green curve in Fig. 3A) (*13*). These LR-DFT simulations entirely omit electron correlations and exclusively inform about the static mean-field scattering (independent particle emission) delay. By comparing the LR-TDDFT and LR-DFT simulations with the experimental results, the relative contribution of large-scale electron-correlation–driven collective versus the nonresonant mean-field dynamics can be uncovered. For the concerned energy range, there is a substantial difference in terms of relative streaking delay between the correlated and the mean-field emission cases. This difference increases in the lower photoelectron energy region.

To test the reproducibility of our findings, the measurements have been repeated with a different attochirp, which has been estimated to be 7500 as^2^. The results of these measurements are displayed in Fig. 3B and reproduce the curves reported in Fig. 3A. The related streaking delays show a very good agreement with the correlated theoretical calculations, which include the new attochirp. Also in this case, the independent particle model underestimates the experimental data. It is worth mentioning that performing measurements with different attochirp values also allowed to identify the role of the chirp in the raw data. In particular, we could confirm that the presence of negative values at energies larger than 12 eV observed in Fig. 3A predominantly arises from the XUV intrinsic chirp, indeed almost vanishing in Fig. 3B (see fig. S1 for further details). Instead, the photoemission delay induced by the GPR remains strictly positive.

## DISCUSSION

The results reported in Fig. 3 (A and B) already suggest that the electron correlations play a crucial role in the photoemission delay around the GPR in C_60_. The contribution of the pure plasmonic correlation can then be quantified by subtracting the LR-DFT curve from the LR-TDDFT curve (including the CTMC results), as shown in Fig. 3C. Because all propagation effects are identical in the correlated and independent particle model, the result of the subtraction can directly be visualized in terms of the EWS delay instead of a measured streaking delay. We note that this quantity is independent of the attochirp by definition (see notes S3 and S4). The resulting curve represents the correlation-driven photoemission delay, i.e., the EWS delay originating exclusively from the large-scale correlation-induced collective excitation of the GPR. It ranges from a minimum of 50 as to about 300 as in the lower energy region.

The LR-TDDFT model also elucidates the fundamental origin of this delay time. The calculations indicate that the collective excitation is responsible for the creation of a correlation-induced scattering potential. On the one hand, the imaginary part of the potential shows a broad local minimum near the GPR energy (inset of Fig. 3C). This correlation-induced attractive shape results in a transient trapping of the photoelectron and thus in a positive photoemission delay (*31*). On the other hand, the real part of this potential blocks (screens) the radiation at energies below the GPR peak to favor the resonance growth, while above the GPR peak, it allows (anti-screens) the radiation to couple with the plasmon, therefore inducing its decay. In this framework, screening induces longer delay, as it is evident at lower photoelectron energy in Fig. 3B. Anti-screening, instead, causes efficient decay and therefore relatively faster emission or shorter delay at higher photoelectron energy.

In conclusion, we used attosecond photoemission chronoscopy to directly access the fundamental mechanisms of the plasmonic response in the fullerene C_60_. We found that the delay accumulated by the electron in the plasmonic potential is dominated by quantum mechanical correlations. We measured it to range from 300 to 50 as in the kinetic energy range between 8 and 24 eV. With the support of combined quantum and classical simulations, we also demonstrated that in this size regime, an ensemble of incoherent single-particle excitations is not enough to describe the scenario, but the large-scale correlation plays the pivotal role in shaping plasmon excitation. The excellent agreement of the measured C_60_ data with LR-TDDFT theory suggests that the theoretical model captured the large-scale correlations that are the essence of quantum plasmon dynamics for ultra-small systems. We note that our findings do not completely exclude the contribution of long-lived lines, such as autoionizing states, to the GPR as previously discussed. However, attosecond streaking spectroscopy is also sensitive to narrowband components. In particular, the emission from long-lived states is imprinted in the streaking spectrogram as flat photoelectron bands, i.e., without showing XUV-IR delay dependence in a delay range close to the temporal overlap between the two pulses. In our measurements (Fig. 2, B and C), we did not observe any clear signature of these contributions, thus suggesting that narrowband components are at least not dominating channels in the plasmonic response of C_60_.

Given the importance of plasmonics in many fields of science, our study contributes to understand the fundamental mechanisms of plasmons in the extreme conditions of subnanometer systems. This knowledge can be particularly important for the future development of novel technology involving quantum plasmonics. For example, even if the ultrafast relaxation of ultra-small particles might impose somewhat of a practical challenge for quantum light technology (e.g., single-photon sources), they can enhance the efficiency of plasmon-induced electronic processes, such as in catalytic reactions or as controllable nanoscale slow-electron sources (*7*).

## MATERIALS AND METHODS

### Experimental methods

The work included data from two experimental campaigns, in which two different values of attochirp were used. For the first measurement set, a commercial driving laser system (Femtopower, Spectra Physics) was used that produced CEP-stabilized pulses at 800 nm with 6 mJ energy per pulse and 25-fs duration at 1-kHz repetition rate. Single-shot CEP fluctuations were within ∼200 mrad (root mean square). For the second measurement set, a different Ti:sapphire laser system has been used (Legend DUO HE+, Coherent) delivering 10-mJ pulses [pulses (6 mJ) were used for the experiments], with 25-fs pulse duration, at 800-nm central wavelength and 1-kHz repetition rate. The CEP was actively stabilized, with residual fluctuations <300 mrad. For both campaigns, the laser pulses were compressed by combining a helium-filled hollow-core fiber and a chirped-mirror compressor. In this way, sub–5-fs NIR pulses were obtained. Isolated attosecond pulses were generated in the XUV spectral range between 15 and 35 eV (see fig. S1A) through polarization-gated HHG (*27*, *32*, *33*). In the first campaign (attochirp ~12,500 as^2^), HHG was driven in a pulsed valve, while in the second one (attochirp ~7500 as^2^) HHG was produced in a semi-infinite gas cell. Isolated gas-phase C_60_ molecules were delivered into the interaction region by sublimating C_60_ powder in a dedicated oven at about 550°C. During the neon streaking runs, the C_60_ jet was blocked by an electronically controlled shutter, which enabled a fast switching between targets without cooling down the oven. Neon was delivered into the chamber through a needle valve, allowing the fine adjustment of gas pressure in the interaction volume and also to switch the rare gas target injection on and off. To preserve the mutual synchronization, the spectrograms for C_60_ and neon were acquired in short interleaved intervals. In particular, the experiment was performed by switching between the two targets every 1 min for each delay. Further details are explained in the Supplementary Materials.

### Theoretical methods

A DFT approach was used to construct the ground state electronic structure of C_60_ and a LR-TDDFT method was deployed to describe the photoabsorption (*13*). LR-TDDFT incorporates many-body correlations between the electrons self-consistently. The Leeuwen-Baerends exchange correlation functional was implemented to produce accurate asymptotic behavior of ground and excited/continuum electrons. LR-TDDFT calculates the dynamical response of the system to the external dipole perturbation by photoabsorption. The photoionization amplitude driven by an XUV pump for the transition from a bound to a continuum state involves complex induced potential that includes electron correlations needed to describe the giant plasmon. The energy derivative of the phase of this amplitude provides the intrinsic EWS time-delay of the emission. The CTMC was implemented to simulate the propagation of these ionized electrons within different electrodynamic fields. For the single-photon ionization, the ionization is in the linear-response regime and the probability is solely dependent on the XUV spectral intensity. Thus, the LR-TDDFT absorption cross sections were used. Photoelectrons were then classically propagated in the electric fields by solving the differential equations of motion using the Runge-Kutta-method. In the propagation, three fields are considered: (i) A static Coulomb field after ionization due to the singly charged residual target which is assumed instantaneous after the electron-hole delocalization. (ii) Around the highly polarizable C_60_, the streaking laser field induces an enhanced near-field that shakes the delocalized electron cloud. (iii) The streaking near-infrared (NIR) field is responsible for the momentum shift of the ionized electron distribution. The effect of the chirp of broadband XUV pulses and the CLC causing additional delays were considered in CTMC. Thus, in addition to the EWS delay, the delay contributions by all these effects are separately captured. Further details are explained in the Supplementary Materials.

## Supplementary Materials

### This PDF file includes:

Supplementary Text Figs. S1 to S7 References
