# Supplementary material for: Correlation-driven attosecond photoemission delay in the plasmonic excitation of C60 fullerene
Source: Sci Adv. 2025 Feb 12;11(7):eads0494. doi: 10.1126/sciadv.ads0494 (PMC11818021; doi:10.1126/sciadv.ads0494)
Supplement: Supplementary file 1 — Supplementary Text Figs. S1 to S7 References [file sciadv.ads0494_sm.pdf]

Science Advances
AAAS

Supplementary Materials for

Correlation-driven attosecond photoemission delay in the plasmonic excitation of C_{60} fullerene

Shubhadeep Biswas *et al.*

Corresponding author: Francesca Calegari, francesca.calegari@desy.de; Matthias F. Kling, kling@stanford.edu; Himadri S. Chakraborty, himadri@nwmissouri.edu

Sci. Adv. **11**, eads0494 (2025)
DOI: 10.1126/sciadv.ads0494

This PDF file includes:

Supplementary Text
Figs. S1 to S7
References

Experimental procedure

The work included two sets of measurements performed at Politecnico di Milano with different XUV generation setups. For the first set, the laser setup and the attosecond beamline are described in detail in Seiffert et al. [27]. In brief, a commercial driving laser system (Femtopower, Spectra Physics) was employed, that produced carrier envelope phase (CEP)-stabilized pulses at 800 nm with 6 mJ energy/pulse, 25 fs duration at 1 kHz repetition rate. Single-shot CEP fluctuations were within ~200 mrad (rms). The laser pulses were further compressed by combining a helium-filled hollow-core fiber and a chirped-mirror compressor. In this way, sub-5-fs near-infrared (NIR) pulses were obtained, with 2.5 mJ energy/pulse and a central wavelength around 720 nm. The above-mentioned pulses were employed to seed the attosecond beamline that consists of a two-arm pump-probe interferometer. In one arm, isolated attosecond pulses were generated in the extreme ultraviolet (XUV) spectral range between 15 and 35 eV (see Fig. S1A), by employing polarisation-gated [27,32,33] high-order harmonic generation (HHG). In the second arm, the NIR pulses, propagated through a piezoelectric delay stage, were used as the streaking field in the experiment. The opportunely delayed NIR pulses and the attosecond pulses were recombined through a drilled mirror and focused into the interaction chamber by a toroidal mirror at grazing incidence. The interferometer was actively stabilized by employing an auxiliary He-Ne continuous-wave laser, allowing for a delay stability of around 20 as rms during the acquisition of each streaking spectrogram. After the interaction chamber, the XUV spectrum of the attosecond pulses was measured with a high-resolution reflective-grating-based XUV spectrometer during each measurement in real time.

For the second set of measurements, a different Ti:sapphire laser system has been used (Legend DUO HE+, Coherent) delivering 10-mJ pulses (6-mJ pulses were used for the experiments), with 25-fs pulse duration, at 800-nm central wavelength and 1-kHz repetition rate. The carrier-envelope phase (CEP) was actively stabilized, with residual fluctuations <300 mrad. The pulses were first compressed using a 3-m-long stretched hollow capillary fiber (HCF) with pressure gradient filled with helium (1.8 bar output pressure). A pulse energy of 3 mJ was obtained at the output. After 12 reflections onto the chirped mirrors (PC1332 from Ultrafast Innovations), pulses as short as 4 fs were achieved. Pulse duration was measured by D-scan.

The visible/near infrared (VIS/NIR) beam was divided into two parts using a beam splitter. The reflected portion of the beam was focused by a mirror with a focal length of 0.75 m into a semi-infinite gas cell filled with krypton. Isolated attosecond pulses were produced by employing polarization gating technique. A 150-nm-thick aluminum filter was used to filter out the fundamental radiation and the low order harmonics. The resulting spectrum spans from 15 to 35 eV. The temporal duration of the XUV pulses (210 ± 20 as) was measured by reconstruction of a streaking trace obtained in Argon, using the ePIE algorithm [36,37]. This is the experimental condition of attosecond pulse generation for which an attochirp value of 7500 as^2 was extracted by fitting the streaking delays in Ne (see the paragraph about XUV chirp).

For both sets of measurements, the following laboratory frame is defined: z is the laser propagation axis, y is the polarization axis of XUV and NIR fields. x in the axis perpendicular to the y and z.

Isolated gas-phase C_{60} molecules were delivered into the interaction region by sublimating C_{60} powder in a dedicated oven at about 550° C. The heater was enclosed within a cooling jacket to reduce thermal load on the vacuum setup and dark counts on the detector. The jacket also

acted as the skimmer for the effusive jet of C_{60} molecules which came out of the heater assembly through a 2-mm wide opening. During the neon streaking runs, the C_{60} jet was blocked by an electronically controlled shutter, which enabled a fast switching between targets without cooling down the oven. Neon was delivered into the chamber through a needle valve allowing the fine adjustment of gas pressure in the interaction volume and also to switch the rare gas target injection on and off.

The photoelectrons produced by the XUV light and streaked by the NIR field were detected by a velocity-map-imaging (VMI) spectrometer, where electrons were projected onto the micro-channel plate detector (Y-Z plane). The detection plane was defined by laser polarization and propagation directions. To record the streaking spectrograms, integrated counts of electrons emitted within the angular region of 50° along the polarization direction were considered as a function of the XUV-NIR pulse delay. The VMI data was not Abel-inverted to avoid a reduction of the signal-to-noise ratio. For this reason, the experimental spectrograms were constructed by considering the projected photoelectron spectra as a function of the projected energy

$$E_{pol} = \frac{p_{pol}^2}{2m} \qquad (1)$$

where p_{pol} is the momenta of the detected electron along laser polarization direction, and m is the electron mass. We note here, that electron spectra obtained theoretically were also projected from the 3D distributions and obtained in the same fashion.

In our experiments the streaking delay around the giant plasmon resonance of C_{60} is measured in reference to the neon 2p electron emission. Since the photoelectron spectra under these conditions for the two targets are not energetically separated, the streaking measurements in C_{60} and neon were done sequentially. In order to preserve the mutual synchronization, the two spectrograms were acquired in short interleaved intervals. In particular, the experiment was performed by switching between the two targets every 1 minute with a short waiting time in between, so the spectrograms for the two targets were acquired in close sequence for each delay step. Additional measurements were taken to verify that the waiting period when switching target was sufficient to ensure minimal influence of one target onto the other's streaking data. Fig. S1B shows that the background signal level achieved is the same regardless of which the preceding sample was, and an order of magnitude lower than with either target injected.

Each streaking spectrogram was acquired in a delay range of around 10 fs around the peak amplitude of the NIR pulse, with a total acquisition time of around one hour for a single complete scan. Due to the angular integration around the polarization direction, a single scan yields, for each target, two spectrograms corresponding to the up and down directions along the vertical polarization vector. For the two sets of measurements, we performed eight independent measurements in total.

Extraction of relative streaking delays

To quantify the streaking delay as a function of the projected photoelectron energy, E_{pol}, the relative phases of the streaking oscillations at different photoelectron energy regions were evaluated. The first step is to extract the streaking contour values of E_{pol}. As the entire delay

range of a given streaking spectrogram consists of about three optical cycles, a single optical cycle was randomly chosen (random starting delay value) and a constant signal threshold was followed over the full delay range at different projected electron energies separately. As electrons for each central projected electron energy are streaked by the instantaneous vector potential of the NIR field, the constant threshold contour would follow a trace at higher as well as lower energies in an oscillatory pattern (c.f. Fig. 2, B and C). The extracted streaking contour lines were then fitted with a function which resembles the shape of the NIR waveform:

$$E_{pol}(t) = A\, e^{-\left(\frac{t-t_0}{\sigma}\right)^2} sin(\omega t - \varphi) + E_0 \tag{2}$$

For fitting of the contour lines for a specific central projected electron energy, A, E_0 and φ were kept as free fitting parameters, whereas other parameters such as t_0, σ and ω were assumed to be constant for all energies. A similar analysis technique was also used in Ref. [27]. For each random sample (optical cycle), the error, which also determines the weight of the sample, in the streaking delay measurement is governed by the confidence interval of φ obtained from the fitting. However, the random selection was done for statistically significant number of times, providing weighted mean and weighted standard deviation of the distribution of the streaking delays. The final results (c.f. Fig. 3A) were obtained by weighted averaging over all the results obtained from five independent measurements.

It is to be noted that the above procedures were followed for individual C_{60} and neon runs in the same fashion providing relative delays between the two targets. For individual spectrograms and for each random sample the projected-energy dependent streaking delay is defined by:

$$\tau_s(E_0) = \frac{\varphi}{\omega}, \tag{3}$$

where the absolute timing is not known. With two targets, however, their relative streaking delay can be determined:

$$\tau_{s,rel}(E_0) = \tau_{s,C_{60}}(E_0) - \tau_{s,Ne}(E_0), \tag{4}$$

which does not depend on the choice for the zero delay.

Due to the relatively large gap between the first ionization energies of C_{60} (7.6 eV) and neon (21.6 eV), the photoelectrons produced from C_{60} are expected to reach higher kinetic energies than that from neon. In such high-energy region for neon, the relative streaking delay cannot be reliably extracted from the raw data with the above-mentioned method. For this reason, the streaking delay in neon for photoelectron energies above 18 eV was extrapolated. To do so, we used a fitting function which captures the effect of Coulomb-laser coupling (CLC) [29] modified by the chirp of the XUV spectrum:

$$\tau(E) = a\left[\frac{Z}{(2E)^{\frac{3}{2}}}(1 - ln(0.37ET_{NIR}))\right] + \tau_0, \tag{5}$$

where Z is the charge state of the residual ion, E is the kinetic energy of the electron, and T_{NIR} is the pulse duration of the streaking NIR pulse [29]. Here a and τ_0 are the fitting parameters, which represent the effect of XUV chirp and the arbitrary time offset, respectively. The choice of this function is supported by the fact that the streaking delay for neon 2p electron emission is dominated by CLC in the higher energy region, so the EWS delay of the Ne target can be neglected.

Semi-classical trajectory simulations

To understand the experimental observations, we performed semi-classical simulations. This includes quantum mechanical calculations based on linear-response time-dependent density-functional approximation (LR-TDDFT) which describes the XUV plasmon excitation including electron correlations among different electronic states. To describe the propagation of electrons in the laser-induced near field of the highly polarizable C_{60}, we used the near-field for C_{60} reported in Refs. [30,38] employing time-dependent density functional theory (TDDFT). Additionally, we obtained the near-field independently from classical electromagnetic (CEM) theory describing the C_{60} as a simple spherical dielectric shell. The ionized electrons were then classically propagated within the combined laser and induced near-field. This was numerically performed with classical trajectory Monte-Carlo (CTMC) simulations. This simulation also considers the effect of the broadband chirp in the XUV pulse which ionizes the target. Finally, our simulations mimic the experimental detection scheme by projecting the final momenta of the electrons. All the major parts of the simulation are described in detail below.

Quantum LR-TDDFT calculations for EWS delay

The XUV ionization step is included in these simulations via LR-TDDFT which provided partial ionization delays (Fig. S2, A and B) and partial cross sections (Fig. S2, C and D) for different electronic states of C_{60}. For neon, ionization from its 2p level is considered.

Ground state theory:

The details of the method follow the density functional theory (DFT) framework in the spherical geometry as described in Ref. [13]. The C_{60} molecule is modelled by smearing the total positive charge of 60 C^{4+} ions into a spherical jellium shell, fixed in space. This utilized an experimentally known C_{60} mean radius ($R = 3.54$ Å) and a width ($\Delta = 1.3$ Å) determined *ab initio [39]*. Inclusion of molecular orientations will have minimal effect on the result due to the C_{60} symmetry [40,41]. The delocalized system of total 240 valence electrons from 60 carbon atoms ($2s^2 2p^2$ of each atom) constructs the ground state in the Kohn-Sham frame as follows.

Using the single-particle density $\rho(r)$ the ground state DFT potential can be written as,

$$V_{DFT}(\boldsymbol{r}) = V_{jel}(\boldsymbol{r}) + \int d\boldsymbol{r}' \frac{\rho(r')}{|\boldsymbol{r}-\boldsymbol{r}'|} + V_{XC}[\rho(\boldsymbol{r})], \quad (6)$$

where the 2nd and 3rd terms on the right are the direct and the exchange-correlation (xc) components. One part of V_{XC} is parametrized directly from $\rho(\mathbf{r})$ by the following formula [42]:

$$V_{XC}[\rho(\boldsymbol{r})] = -\left(\frac{3\rho(r)}{\pi}\right)^{\frac{1}{3}} - 0.0333\, log\left[1 + 11.4\left(\frac{4\pi\rho(r)}{3}\right)^{\frac{1}{3}}\right], \quad (7)$$

in which the first term on the right is exactly derivable by a variational approach from the Hartree-Fock (HF) exchange energy of a uniform electron system with a uniform positively charged background and the second term is the so-called correlation potential, a quantity not borne in HF formalism. The other part of xc-functional further refines the model by adding a parametrized potential [43] in terms of the reduced density and its gradient $\nabla\rho$ as follows,

$$V_{LB} = -\beta[\rho(\boldsymbol{r})]^{\frac{1}{3}} \frac{(\xi X)^2}{1+3\beta\xi X\, sinh^{-1}(\xi X)}, \quad (8)$$

where $\beta = 0.05$ is empirical and $X = [\nabla\rho]/\rho^{4/3}$. The parameter ξ is a factor arising in transition from the spin-polarized to spin-unpolarized form [44]. This method of gradient-correction to the xc-functional, termed as LB94, leads to a considerable improvement in the asymptotic behaviour of the electron by comparing well with the exact Kohn-Sham potentials calculated from correlated densities. Indeed, for the optical spectra, the LB94 type approach performed very well for Silicon clusters [45].

The ground state C_{60} orbitals are obtained by solving the Kohn-Sham equation with the potential Eq. (6). This model produced HOMO and HOMO-1 to be of $2h$ ($l = 5$) and $2g$ ($l = 4$) character respectively with each having a radial node – a result known from the quantum chemical calculation [46] supported by direct and inverse photoemission spectra [47], and from energy-resolved electron-momentum density measurements [48].

Photoionization and time delay theory:

Choosing the photon polarization along the z-axis, the photoionization dipole transition amplitude in a single-channel independent-particle (IP) approximation, which omits electron correlations, is:

$$d(\boldsymbol{k}) = \langle\psi_{\boldsymbol{k}l'}|z|\phi_{nl}\rangle. \quad (9)$$

Here $\boldsymbol{k}$ is the momentum of the continuum electron, z is the one-body dipole operator, ϕ_{nl} is the bound wavefunction of the target obtained in the previous section, and the outgoing spherical continuum wavefunction $\psi_{\mathbf{k}l'}$ is

$$\psi_{\boldsymbol{k}l'}(\boldsymbol{r}) = (8\pi)^{3/2} \sum_m e^{-i\eta_{l'}} R_{kl'}(r) Y_{l'm}(\boldsymbol{\Omega_r}) Y^*_{l'm}(\boldsymbol{\Omega_k}), \quad (10)$$

with $l' = l \pm 1$. In Eq. (10), the scattering phase $\eta_{l'}(k)$ contains contributions from both short-range and Coulomb potentials, besides a constant phase $l'\pi/2$, and $R_{kl'}$ is the radial continuum wave. This accounts for the mean-field effects.

The linear response time-dependent DFT (LR-TDDFT), used here to calculate the *full* transition amplitude, includes many-electron effects and utilizes the advanced Green's function G [49-51]. The ensuing photoionization amplitude formally reads

$$D(\boldsymbol{k}) = \langle \psi_{\boldsymbol{k}l'} | \delta V^*(\boldsymbol{r}) + z | \phi_{nl} \rangle = d(\boldsymbol{k}) + \langle \delta V^*(\boldsymbol{r}) \rangle. \quad (11)$$

Here $\delta V(\mathbf{r})$ is the energy-dependent complex induced potential that accounts for electron correlations up to the linear terms of the many-body perturbation theory. In LR-TDDFT, $z + \delta V(\mathbf{r})$ is proportional to the induced frequency-dependent changes in the electron density [52]. This change is

$$\delta\rho(\boldsymbol{r}'; \omega) = \int \chi(\boldsymbol{r}, \boldsymbol{r}'; \omega) z d\boldsymbol{r}, \quad (12)$$

where the full susceptibility χ builds the dynamical correlation from the independent-particle DFT susceptibilities

$$\begin{aligned} \chi^0(\boldsymbol{r}, \boldsymbol{r}'; \omega) \quad = \quad & \sum_{nl}^{occ} \phi_{nl}^* (\boldsymbol{r}) \phi_{nl}(\boldsymbol{r}')\; G(\boldsymbol{r}, \boldsymbol{r}'; \epsilon_{nl} + \omega) \\ + \quad & \sum_{nl}^{occ} \phi_{nl} (\boldsymbol{r}) \phi_{nl}^*(\boldsymbol{r}')\; G^*(\boldsymbol{r}, \boldsymbol{r}'; \epsilon_{nl} - \omega)' \end{aligned} \quad (13)$$

through the matrix equation $\chi = \chi^0[1 - (\partial V/\partial\rho)\chi^0]^{-1}$ involving the variation of the ground-state potential V with respect to the ground-state density ρ. The radial components of the full Green's functions in Eq. (13) are constructed with the regular (f_L) and irregular (g_L) solutions of the homogeneous radial equation

$$\left(\frac{1}{r^2}\frac{\partial}{\partial r} r^2 \frac{\partial}{\partial r} - \frac{L(L+1)}{r^2} - V_{DFT} + E\right) f_L(g_L)(r; E) = 0, \quad (14)$$

as

$$G_L(r, r'; E) = \frac{2 f_L(r_<; E) h_L(r_>; E)}{W[f_L, h_L]}, \quad (15)$$

where W represents the Wronskian and $h_L = g_L + i\, f_L$.
Using Eq. (10) in Eq. (11), the LR-TDDFT amplitude takes the explicit form [50]

$$\begin{aligned} D(\boldsymbol{k}) \quad = \quad & (8\pi)^{3/2} \sum_m C_{l'm}\, e^{-i\eta_{l'}} Y_{l'm}^*(\boldsymbol{\Omega_k}) \\ & \times \langle R_{kl'} | r + \delta V^*(r) | R_{nl} \rangle' \end{aligned} \quad (16)$$

where R_{nl} is the radial bound wavefunction and $C_{l'm}$ includes the angular momentum Wigner coefficients.
The radial component $R_{kl'}$ of the final continuum wavefunction $\psi_{\boldsymbol{k}l'}$ has the appropriate asymptotic behavior:

$$\begin{aligned} \lim_{r\to\infty} R_{kl'}(r) \sim & \lim_{r\to\infty} [cos(\delta_{l'})\, f_{l'}(kr) \; + \; sin(\delta_{l'})\, g_{l'}(kr)] \\ & = sin\,(kr - \tfrac{1}{2}\pi \; + \tfrac{z}{k} ln(2kr) + \; \zeta_{l'} \; + \; \delta_{l'}), \end{aligned} \quad (17)$$

where ζ and δ are respectively the Coulomb and short-range phase-shifts seen by the ejected electron.

The nl subshell cross section $\sigma_{nl} = \sum_{l'} \sigma_{nl\to kl'}$, where $\sigma_{nl\to kl'}$ is proportional to the modulus square of Eq. (16), integrated over the photoelectron direction $\Omega_{\mathbf{k}}$. LR-TDDFT previously predicted [39] oscillatory photoemission cross sections of HOMO and HOMO-1 in C_{60} at energies higher than plasmon resonance energies which agreed well with the experiment [53] and with quantum chemical calculations [53,54]. Similar geometry-based oscillations in high-harmonic spectra of icosahedral fullerenes were predicted [40,41]. This points to a common spectral implication between photoionization and recombination processes.

More importantly, LR-TDDFT total cross section also qualitatively explained [13,55] the measured low-energy (giant) and high-energy plasmon resonances at about 20 and 40 eV respectively in C_{60}. In fact, the emergence of plasmon resonances can be thought of as originating from the formation of collective states under the influence of external electromagnetic field. Over the giant plasmon resonance region, the interplay between the real and imaginary components of $\delta V^*(r)$ further uncovers the critical role of the collective electron correlations that drives the plasmon dynamics [56]. As a function of energy, the real component is found to slice through a zero at 20 eV, the energy of the resonance peak. Consequently, at energies from below to above this peak, the real component switches from negative to positive values. This represents, respectively, the mode-switch from “screening" to “anti-screening" of the incoming radiation. On the other hand, as characteristics of the collective electron motions [56], the imaginary component (c.f. Fig. 3C inset) shows an attractive well with its minimum at the resonance peak. Since around the plasmon peak the real and imaginary component are, respectively, weak and strong, one may visualize that the induced field oscillates 90° out-of-phase with the driving XUV field [49]. Hence, the strong imaginary component dominates the matrix element to drive plasmonic enhancement. This energy-transient attractive field developed as part of the plasmon dynamics results in capturing electrons to populate the collective state leading to a subsequent delay in the intrinsic photoemission time.

The angle-resolved Eisenbud-Wigner-Smith (EWS) time delay is associated with the phase of Eq. (16) for emissions at a solid angle Ω_k [57]. In a non-angle-resolved measurement the total amplitude is directly close to the $\Omega_{\mathbf{k}}$-integrated dipole matrix elements. Thus, following Ref. [20], the LR-TDDFT nl ionization phase can be approximated by

$$\Gamma_{nl} = arg\left[\sum_{l'} \sqrt{\sigma_{nl\to kl'}} exp(i\Gamma_{nl\to kl'})\right], \tag{18}$$

where $\Gamma_{nl\to kl'}$ are the phases of corresponding amplitude [Eq. (16)], ignoring the spherical harmonics. Hence, these channel phases are the sum of the scattering phase η and the phase of the radial matrix element. The energy differential of Γ_{nl} is the EWS time delay of the emission from the nl subshell [21,22]. Equation (18) and the delay derived from it previously explained $3p$ photorecombination measurements very well. For such studies, the reconstruction of attosecond beating by interference of two-photon transitions (RABBITT) method was employed [20]. Thus, LR-TDDFT directly provides the EWS delays with the large-scale correlation of the plasmon resonance; the contribution to the phase from single-electron resonances is largely smoothened out. Obviously also, omitting δV^* in Eq. (16) provides EWS delays without correlations, the mean-field delay, in the LR-DFT scheme. Thus, this delay is evaluated from only the phases ζ and δ in Eq. (17).

Subsequently, another approximation scheme is deployed to extract the total emission time delay as a function of the photoelectron kinetic energy (PEKE) (including the projection along the laser polarization axis) which is required to enable the comparison with the present streaking measurements. This has been carried out by expressing the nl subshell cross sections and delays as functions of PEKE. The total delay for a given value of PEKE is then determined by averaging, that is, adding the delays weighted by the corresponding subshell cross section fractions (the ratios of subshell to total cross sections). Since each level has different ionization thresholds, the plasmon resonance structures occur at different PEKE values for each level in resulting EWS delays, see Fig. S2A. This results in some smaller structures in the total EWS delay as a function of PEKE, although the effect of GPR is dominating due to its spectacularly higher emission strength.

Following [52], the plasmon phenomenon can be visualized by the Fano formalism [58] which includes the effect of interchannel coupling between the channel wave functions $\Psi_{nl}(E)$ of LR-DFT matrix elements d_{nl}. This recasts the LR-TDDFT matrix elements D_{nl}, Eq. (6), for a photon energy E as

$$D_{nl}(E) = d_{nl}(E) +$$

$$\sum_{n'l' \neq nl} \int dE' \frac{\left\langle \Psi_{n'l'}(E') \middle| \frac{1}{r_{nl} - r_{n'l'}} \middle| \Psi_{nl}(E) \right\rangle}{E - E'} d_{n'l'}(E'), \tag{19}$$

where the sum is over all of the photoionization channels except the nl channel and we consider the principal value for the integral in Eq. (19). The fact that each of nl bound state orbital of C_{60} overlaps strongly with all other C_{60} orbitals ensures that the interchannel coupling matrix elements within the integral will be strong. Further, the existence of plasmons at exactly the same photon energies for all the subshells implies the various LR-DFT matrix elements are "in phase" over these energy regions. Indeed, the band-like nature of the energy levels resulting in close k values likely facilitates this mechanism. Hence, the various terms in the sum of Eq. (14) add up coherently, leading to the dramatic plasmonic increase. Therefore, a large-scale phase-coherent interchannel coupling phenomenology must be responsible for the resonant enhancement in each subshell cross section.

CEM calculations of the fullerene near-field

The response of the C_{60} molecule under the incident electromagnetic wave was modelled considering it as spherical dielectric shell, where the motion of electrons is governed solely by the electric field non-relativistically. This model is adapted following the results given by Andersen and Bonderup [59] and also compared with the time-dependent local-density approximation (TDLDA) results by Maurat et al. [60]. In the limit of $d \ll c/\omega$ (c is the velocity of light and ω is angular frequency of incident radiation), i.e. the extension of the object is much smaller than the reduced wavelength of the radiation, the variation of the incident electric field over the extension of the body is negligible. Then, the incident electric field can be

approximated as uniform electric field $\boldsymbol{E} = E_0\hat{z}$ along the *z* direction. Under this incident field the dielectric sphere gets polarized and distorts the field near the sphere. All these electric fields can be expressed as the negative gradient of a scalar potential, as in electrostatics, and their contributions can be calculated by solving the Maxwell equations along with employing the boundary conditions for $\vec{E}$ and $\vec{D}$, the electric displacement vector.

The symmetry of the problem about the *z*-axis suggests that the electrostatic potential would be a function of r and θ only, and it can be expressed in general as

$$\Phi(r,\theta) = \sum_{n=0}^{\infty}\left(a_n r^n + \frac{b_n}{r^{n+1}}\right) P_n\,(cos\,\theta), \tag{20}$$

where $P_n(\cos\theta)$ are the Legendre polynomials.

In case of C_{60} with inner and outer radii r_1 and r_2, respectively, the potential takes three different forms in three different regions with an asymptotic limit of $\Phi(r \to \infty) = -E_0 r\cos\theta$:

$$\Phi(r,\theta) = -E_i r\,cos\,\theta\,, \qquad r < r_1 \tag{21}$$

$$= \left(Ar + \frac{B}{r^2}\right) cos\,\theta, \qquad r_1 < r < r_2$$

$$= \left(-E_0 r + \frac{p}{r^2}\right) cos\,\theta\,, \qquad r > r_2,$$

where E_i is the screened internal field and p is the induced dipole moment along *z*-axis.

If we apply the conditions of continuity for $\vec{E}$ and $\vec{D}$ we get the coefficients A and B, and E_i as:

$A = \frac{-3\alpha E_0}{(1-\epsilon)(\xi-1)r_2^3}$, $B = \frac{3\alpha\xi E_0}{(1+2\epsilon)(\xi-1)}$ and $E_i = \frac{9\alpha\epsilon E_0}{(1+2\epsilon)(1-\epsilon)(\xi-1)r_2^3}$

with polarizability $\alpha = \frac{p}{E_0} = r_2^3\,\frac{(1-\xi)(\epsilon-1)(2\epsilon+1)}{(\epsilon+2)(2\epsilon+1)-2\xi(\epsilon-1)^2}$ and $\xi = \left(\frac{r_1}{r_2}\right)^3$

The corresponding electric field (Fig. S3, A and B) is given by

$$\tilde{E}(r,\theta) = -E_i\,cos\,\theta\,, \qquad r < r_1 \tag{22}$$

$$= -\epsilon\left(A + \frac{B}{r^3}\right) cos\,\theta, \qquad r_1 < r < r_2$$

$$= -\left(-E_0 + \frac{p}{r^3}\right) cos\,\theta\,, \qquad r > r_2$$

To get the dielectric constant (ϵ), we used the Drude model [61] considering a free electron gas, which gives

$$\epsilon = 1 - \frac{\omega_p^2}{\omega(\omega+i\gamma)}, \tag{23}$$

where $\omega_p = \sqrt{4\pi n_0} = \sqrt{3} r_s^{-\frac{3}{2}}$ is the plasma frequency, $r_s = \sqrt[3]{\left(\frac{r_2^3 - r_1^3}{n_e}\right)}$ for n_e being the number of available electrons, is the Wigner–Seitz radius, and γ is the damping constant. From this above expression it is easy to show that the dynamical response of the system yields two resonances at $\omega_\pm = \omega_p \left(\frac{1}{2} \pm \frac{1}{6}\sqrt{1 + 8\xi}\right)^{\frac{1}{2}}$ and the width of the resonances is determined by the value of γ.

From a very basic definition of absorption cross section as the ratio between the time averages of the rate of energy absorption by the molecule and the incident flux of energy, it can be written as

$$\sigma(\omega) = 4\pi \frac{\omega}{c} Im(\alpha), \qquad (24)$$

where Im stands for imaginary part.

In the present calculations we used an average shell radius as 3.54 Å, whereas 1.3 Å is taken as the shell thickness. The other important quantity for the model is the number of electrons which is 240 in case of C_{60}. The damping parameter γ, whose value is put ad-hoc to match the broad cross section peak, has hardly any influence on the near field and static polarizability calculations when the driving wavelength is in the off-resonant NIR region. The comparison between the radial distributions of the near field obtained from the CEM and the TDDFT calculations [38] are shown in Fig. S3B. The near fields obtained from the two models agree qualitatively well. Qualitatively, because of the slightly different amount of screening effect described by the two models, the screening factor (E_i/E_0) and the maximum field enhancement along the polarization axis differ slightly. Similar comparison with the TDLDA results [60] is also done in the absorption cross section level in Fig. S4A, where it shows overall good agreement between these two, except the narrow peaks given by the TDLDA calculations on the broad structure. Additionally, Fig. S4B shows the behaviour of the real part of the polarizability showing the static and dynamic response of the C_{60} electron cloud under the incident radiation. The values of the electronic static polarizability provided by the CEM and the TDDFT models came out to be 74 Å^3 and 84 Å^3 [38], respectively, both of which are in close agreement with experimentally determined value of 79 ± 4 Å^3 [62].

CTMC simulations

The CTMC simulations start with the ionization step. As being single photon ionization, the ionization probability is solely dependent on the XUV spectral intensity and the absorption cross section of the target. The NIR field does not play a role here. Therefore, the electrons are sampled to the ionization continuum according to the partial ionization cross sections, provided by the LR-TDDFT (LR-DFT to simulate the independent particle case) calculations, of their different initial bound states. The initial kinetic energies of the electrons are $E_{kin} = \hbar\omega_{XUV} - I_{p,k}$, where $I_{p,k}$ is the ionization potential of the involved initial bound state k. These electrons fly apart from the residual ions with an isotropic velocity distribution. The broadband XUV spectrum spans over multiple initial bound states, such that the initial kinetic energy distribution involves a convolution over all the relevant states. In the temporal domain, the electron birth time for every initial state is sampled over the 250 as pulse duration of the XUV pulse assuming a Gaussian pulse shape of XUV laser.

In the spatial domain, the electrons start from the targets according to their respective ground state electronic density distributions. For neon, the 2p ground state density is considered while for C_{60} the used ground state density is calculated by the jellium model within DFT [52]. It reflects the spherical symmetry of the C_{60} molecule whereas in the radial direction it can be approximated by Gaussian distribution centred at the radius (R ≈ 3.54 Å) of the C_{60} cage formed by the carbon atoms (c.f. Fig. 1A). Microscopically, this electron density is formed by the contributions from 2s and 2p electrons from sixty constituent carbon atoms. The 1s electrons are not considered here because these are tightly bound to the atoms. These contributing electrons form three σ and one π bonds adjacent to each carbon atom.

Following the ionization step, the ionized electrons are classically propagated within the electric fields. This is done numerically by solving the differential equations of motion using the Runge-Kutta-method [63-65], that contains an estimate for the error and enables the use of an adaptive step size algorithm. To optimize the calculations, we used finer time steps for the initial part of the propagation where details of different contributing fields are important, whereas in the later stage larger time steps were adapted where electrons move almost as a free particle.

In the propagation step, three kinds of electric field were considered. First, a static Coulomb field is present after ionization due to the singly charged residual target ion. Here, the occurrence of this field is assumed to be instantaneous after the electron hole delocalization. In case of C_{60}, this field is mostly screened within the cage.

Second, around the highly polarizable C_{60} the streaking laser field induces an enhanced near field. Similar to the plasmonic behaviour in metals, the delocalized electron cloud of the C_{60} is driven by the NIR field. At low driving frequencies compared to the resonance frequency of the system, the motion of the electron cloud follows the driving frequency, and the imaginary part of dielectric function vanishes. As a result, within the C_{60} cage the near field almost vanishes due to screening. Outside of the C_{60} cage the near field converges smoothly to the dipolar field going through a maximum near the carbon shell. At far distances the near field strength equals that of the driving laser field. For efficient numerical calculations, the near fields obtained from TDDFT [38] and CEM models were described by analytical functions of the form:

$$\boldsymbol{E}(\boldsymbol{r}) = c_0 \boldsymbol{E_0}, \qquad r < R, \tag{25}$$

$$= c_0 \boldsymbol{E_0} + \left[\sum_{i=1}^{2} \frac{a_i}{e^{((r-b_i)/c_i)} + 1}\right] \left(\boldsymbol{E_0} + \frac{\alpha}{4\pi\epsilon_0} \frac{3(\boldsymbol{E_0} \cdot \hat{r})\hat{r} - \boldsymbol{E_0}}{r^3}\right), r \geq R$$

Here, c_0 depicts the screening whereas the double Fermi function containing a_i, b_i and c_i ensures a smooth transition between the screened part on the inside and the decaying dipole distribution on the outside of the C_{60} cage. The transition point is denoted by R. It should be noted that the above-mentioned differences in the description of the near fields, which stem out from the CEM and TDDFT calculations, hardly influence the final delay results (Fig. S3C). We also note that in the case of neon because of its size and small polarizability, the consideration of the near field enhancement is not required.

Third, the streaking NIR field of intensity between 2 and 5 x 10^{12} W/cm^2, which was determined from the comparison with the high photoelectron energy cutoff of the experimental streaking spectrogram, is responsible for the momentum shift of the ionized electron distribution. It is assumed to be a plane wave as the NIR focus in the experiment is much larger compared to the target dimensions.

To overcome the short cutoff problem of the neon spectrum, discussed above, the CTMC results were also extrapolated in a similar way as done for the experimental results using Eq. (5).

The CTMC simulations intrinsically consider instantaneous XUV ionization of the targets. To include the EWS delays in the final results, we added the state-selective and kinetic-energy-resolved EWS delays obtained from the LR-TDDFT (LR-DFT to simulate the independent particle case) simulations after weighting with their respective partial absorption cross sections. Here, a total of 11 highest lying occupied states have been taken into account: 5 σ states (1h to 1l), having ionization energies ranging from 11.2 eV to 28.5 eV, and 6 π states (2s to 2h), having ionization energies between 7.6 eV and 17.4 eV.

<u>XUV chirp</u>

Finally, the effect of the chirp of the broadband XUV pulses causing an additional spectrally dependent delay of the ionized electrons is fully considered in the CTMC simulations. Here, we obtained the chirp from comparison to the neon 2p emission data only.

In the spectral domain, the XUV pulses can be described by

$$\widetilde{\boldsymbol{E}}(\omega) = \overline{\boldsymbol{E}}(\omega) e^{-i\varphi(\omega)}, \tag{26}$$

Where $\overline{\boldsymbol{E}}(\omega)$ characterizes the spectral amplitude of the XUV spectrum and $\varphi(\omega)$ is the spectral phase. In this description, the spatial structure is assumed to be plane wave because of the larger dimension of the XUV focus compared to the target dimension. The temporal evolution of the field can be obtained by Fourier transformation as

$$\boldsymbol{E}(t) = \frac{1}{2}\frac{1}{\sqrt{2\pi}} \int \widetilde{\boldsymbol{E}}(\omega)\, e^{-i\omega t} d\omega + c.c.. \tag{27}$$

The spectral phase $\varphi(\omega)$ can be described as

$$\varphi(\omega) = \varphi_{CE} + \frac{\beta}{2}(\omega - \omega_0)^2, \tag{28}$$

where φ_{CE} is the CEP of the XUV pulse, which was set to zero, and β is the chirp parameter. Within the CTMC calculations the above-mentioned description of the XUV field enabled us to extract the chirps discussed in Fig. 3A and 3B from the comparison with the corresponding experimental neon data. We note that the negative delays are less pronounced when the chirp is lower (see Fig. 3B). This effect can be easily explained: photoelectrons that have the same kinetic energy for Ne and C_{60} are generated by different photon energies, since the two samples have different ionization potentials. When a positive chirp is present (where photon energy increases with time), the photoelectrons produced in Ne experience a larger delay compared to those generated in C_{60} at the same energy. Thus, the relative delays (C_{60} – neon) exhibit an artificial negative offset caused by the attochirp. By minimizing the attochirp (as seen in Fig. 3B), the offset also decreases. As an important consequence, the zero-crossing around 14 eV

in Fig. 3A predominantly arises from the intrinsic chirp of the attosecond pulse, while the photoemission delay induced by the GPR is strictly positive.

For completeness, Fig. S5 shows all the possible contributions to the relative streaking delays (IR near field, XUV chirp, etc), as discussed above.

In the main text, we explain that the energy-dependent correlated EWS of Fig. 3C is XUV-chirp independent. For completeness, we show in Fig. S6 the comparison of the same quantity extracted for the two different values of attochirp.

Total photo-ionization cross-section of C_{60}

In order to benchmark the calculation of the total photo-ionization cross section (CS) of C_{60}, the CS calculated with LR-TDDFT was compared with the total CS obtained with real-time time-dependent density-functional-theory (RT-TDDFT), i.e., by simulating the full ionization process with time-dependent density functional theory (TDDFT) [66] and t-SURFF [67,68] as implemented in the Octopus code [69]. In the RT-TDDFT simulations, the energy of the incoming photon is assumed to be completely transferred to photoelectrons' kinetic energy, thus the ionization CS is proportional to the photon absorption CS, provided one looks at photon energies in the continuum [70]. We therefore proceeded to calculate the photo absorption CS of C_{60} with our real-time TDDFT approach. In linear response, this amounts to calculating the Fourier transform of the induced dipole moment after an instantaneous kick perturbation [66]. Here, the use of absorbing boundaries is a prerequisite necessary to access the CS in the continuum. In these calculations we used a spherical box of radius 40 a.u. with a 15 a.u. thick CAP with -1 complex amplitude which ensured a good quality in the energy window between 5 to 500 eV [71]. The CS calculated with RT-TDDFT is reported in Fig. S7 and compared with the results obtained with LR-TDDFT. As a further comparison, Fig. S7 also reports experimental CS from literature [16,72], showing a good agreement and benchmarking the LR-TDDFT calculations used in this work.

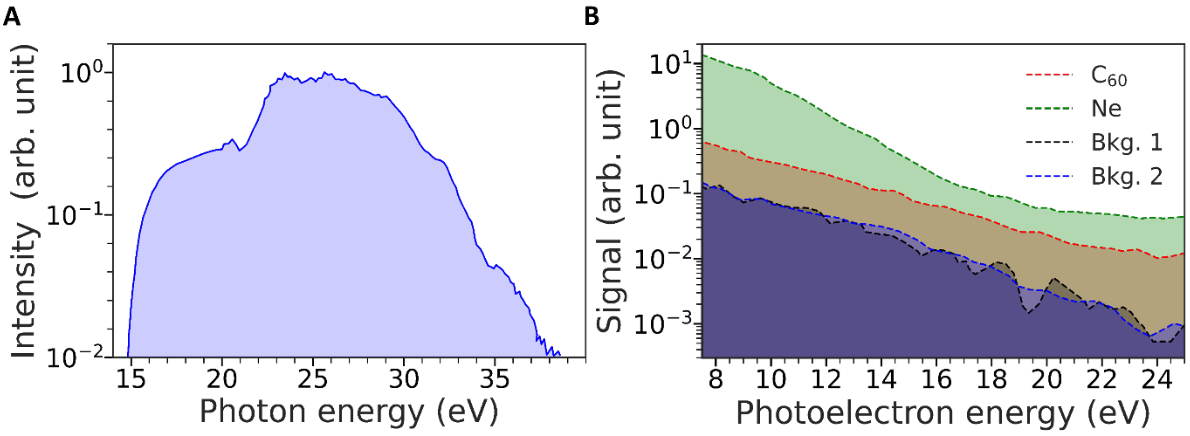

Figure S1: XUV photon spectrum and electron spectra acquired during C_{60}, neon and background runs. (**A**) Energy distribution of the attosecond XUV photon spectrum used in the experiment. The detector acquired

the spectrum down to 20 eV, however the effective XUV spectrum extends to 15 eV, only limited by the transmission of the Al filter. In the figure, the spectrum between 15 and 20 eV only represents the nominal transmission curve of the 150-nm-thick Al foil installed in the beamline. We note that the HHG emission is also expected to decay in this spectral region (15-20 eV) due to phase matching. (**B**) Contrast between electron yields for signal and background runs. The measurements for C_{60} (red) and neon (green) were performed with the respective source switched on, solely. Each measurement was taken in alternation with a background recording where both sources are switched off for a few seconds. Background 1 (black) and 2 (purple) refer to the signal after C_{60} and neon runs, respectively. Both the C_{60} and neon signals are always about an order of magnitude larger than the background in the detected electron energy range.

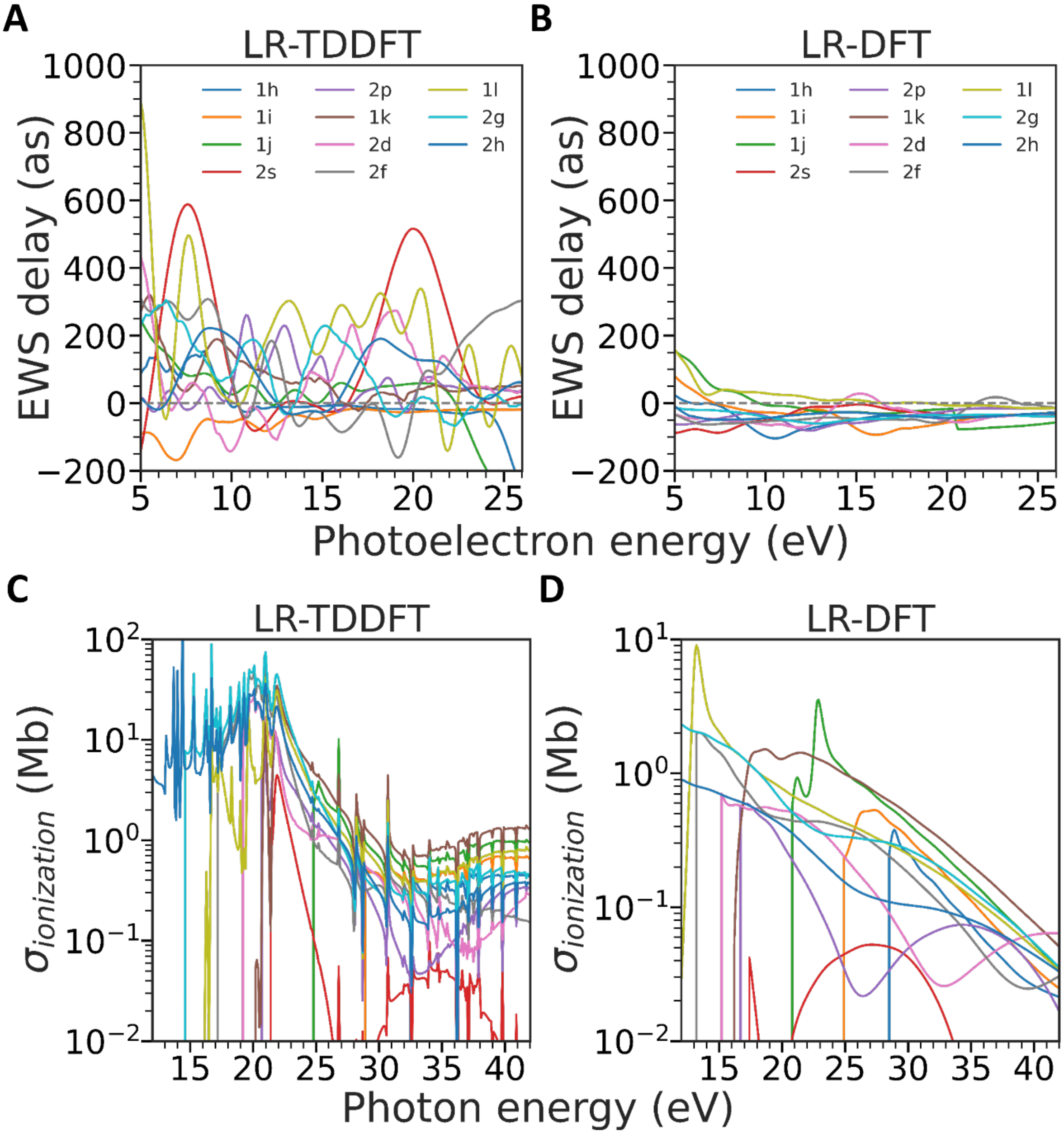

Figure S2: Partial EWS delay and cross sections. (**A**) and (**B**) Partial EWS delays for 11 highest lying subshells (see Fig. 1A) calculated within LR-TDDFT and LR-DFT formalisms, respectively. (**C**) and (**D**) Partial ionization cross sections for these subshells calculated within the above-mentioned formalisms, respectively.

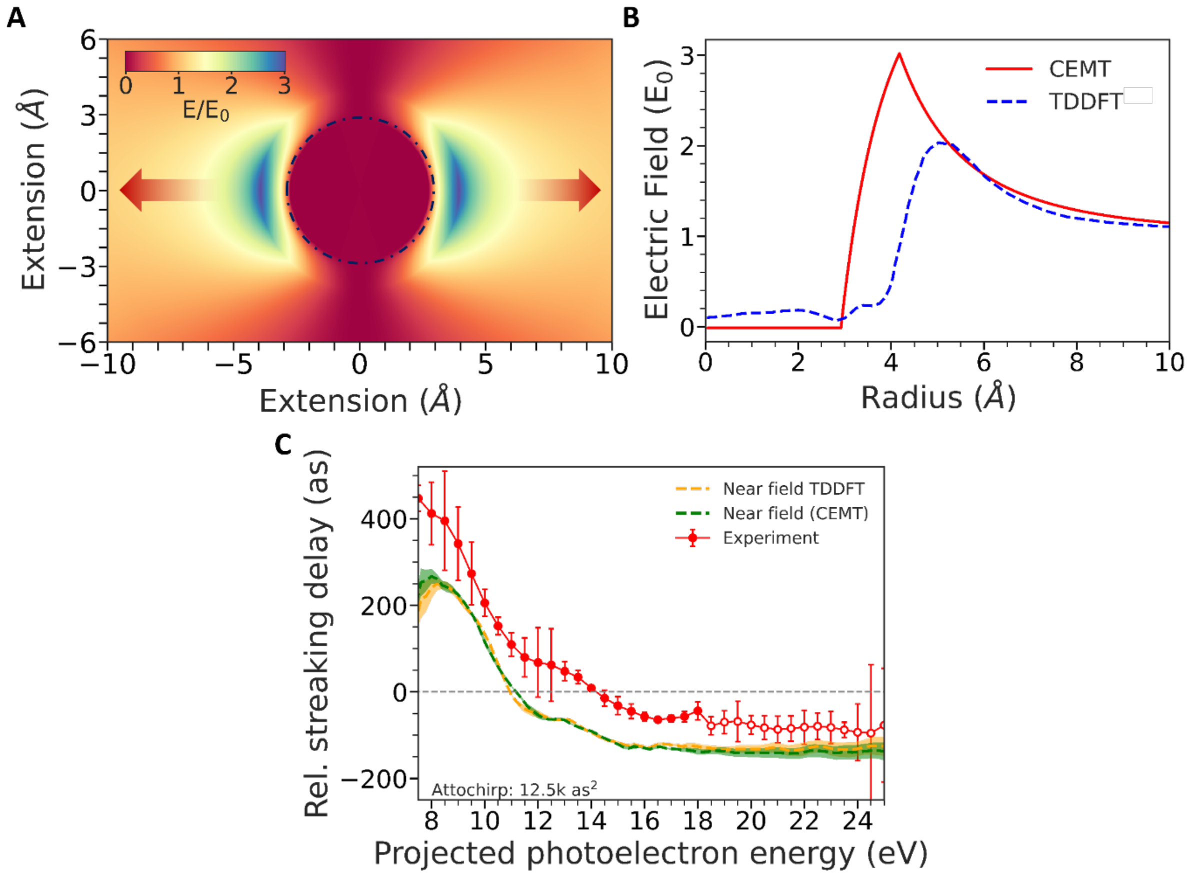

Figure S3: Comparison of effect of near fields calculated with CEM theory and TDDFT. (**A**) The near field distribution of the C_{60} obtained from the classical electromagnetic theory calculations. The circle at the middle represents the extension of the C_{60} cage. The direction of the arrow depicts the polarization direction of the impinging light. Inside the C_{60} cage the field is supressed by screening whereas at the outside it decays as dipolar distribution following a field enhancement near the surface. (**B**) Comparison of effect of near fields calculated with CEM theory and TDDFT [30,38]. (**C**) Comparison of relative streaking delay (only considering XUV chirp, CLC and near field) calculated using nearfield obtained from the two theoretical methods.

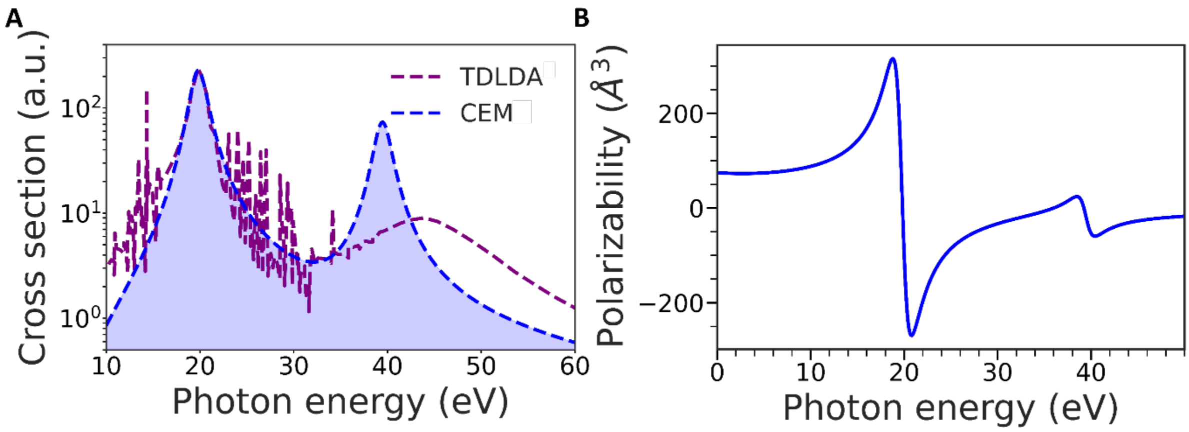

Figure S4: Absorption cross section and polarizability calculated with CEM theory. (**A**) Comparison of absorption cross sections calculated with CEM theory and TDLDA [60]. (**B**) Polarizability of C_{60} as a function of photon energy of the impinging radiation.

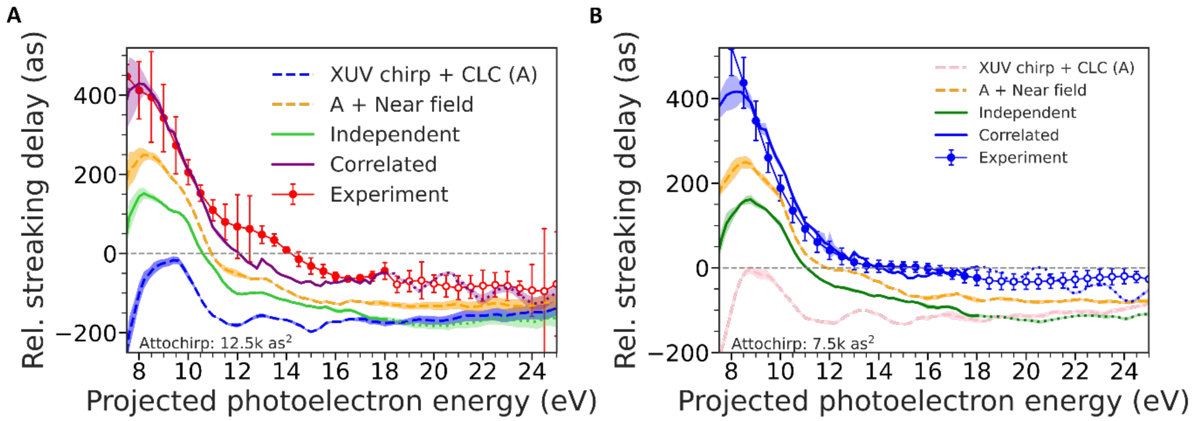

Figure S5: Comparison of different contributions considered in LR-TDDFT simulations. The contributions of XUV chirp and CLC, and also that additionally including the near field are depicted in comparison with the full simulations (Fig. 3 in main text). This calculation has been performed with attochirp of 12500 as^2 (**A**) and 7500 as^2 (**B**).

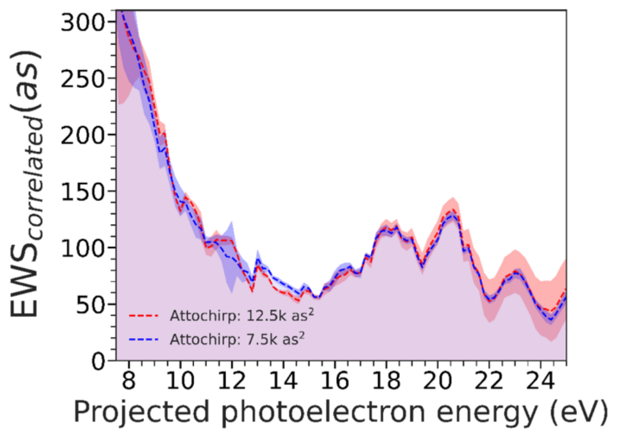

Figure S6: EWS contribution in the presence of different values of attochirp. The EWS delay contribution exclusively from the correlated excitation is extracted from the difference between the results of LR-TDDFT (correlated) and LR-DFT (mean-field) calculations for the two values of attochirp, i.e., 12500 as^2 and 7500 as^2.

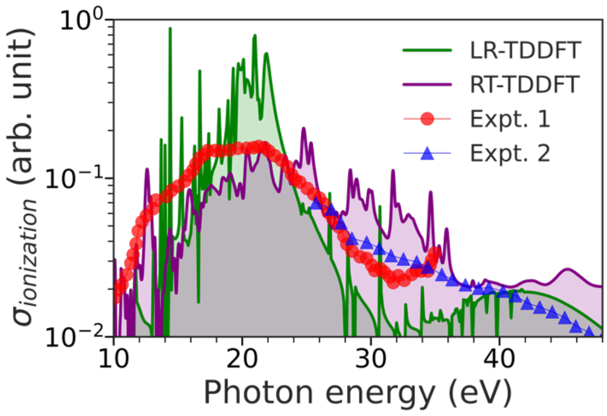

Figure S7: The photoionization cross section as a function of photon energy. The solid curves represent results from TDDFT (LR: linear response; RT: real time) calculations used in this work. These are compared with the experimental photoionization cross sections obtained from Refs. [16] and [72].

REFERENCES AND NOTES

1. A. F. Koenderink, A. Alu, A. Polman, Nanophotonics: Shrinking light-based technology. *Science* **348**, 516–521 (2015).

2. M. I. Stockman, K. Kneipp, S. I. Bozhevolnyi, S. Saha, A. Dutta, J. Ndukaife, N. Kinsey, H. Reddy, U. Guler, V. M. Shalaev, A. Boltasseva, B. Gholipour, H. N. S. Krishnamoorthy, K. F. MacDonald, C. Soci, N. I. Zheludev, V. Savinov, R. Singh, P. Groß, C. Lienau, M. Vadai, M. L. Solomon, D. R. Barton III, M. Lawrence, J. A. Dionne, S. V. Boriskina, R. Esteban, J. Aizpurua, X. Zhang, S. Yang, D. Q. Wang, W. J. Wang, T. W. Odom, N. Accanto, P. M. de Roque, I. M. Hancu, L. Piatkowski, N. F. van Hulst, M. F. Kling, Roadmap on plasmonics. *J. Opt.* **20**, 043001 (2018).

3. K. D. Chapkin, L. Bursi, G. J. Stec, A. Lauchner, N. J. Hogan, Y. Cui, P. Nordlander, N. J. Halas, Lifetime dynamics of plasmons in the few-atom limit. *Proc. Natl. Acad. Sci. U.S.A.* **115**, 9134–9139 (2018).

4. C. Sonnichsen, T. Franzl, T. Wilk, G. von Plessen, J. Feldmann, Plasmon resonances in large noble-metal clusters. *New J. Phys.* **4**, 93 (2002).

5. J. A. Scholl, A. L. Koh, J. A. Dionne, Quantum plasmon resonances of individual metallic nanoparticles. *Nature* **483**, 421–427 (2012).

6. S. Karimi, A. Moshaii, S. Abbasian, M. Nikkhah, Surface plasmon resonance in small gold nanoparticles: introducing a size-dependent plasma frequency for nanoparticles in quantum regime. *Plasmonics* **14**, 851–860 (2019).

7. M. S. Tame, K. R. McEnery, S. K. Ozdemir, J. Lee, S. A. Maier, M. S. Kim, Quantum plasmonics. *Nat. Phys.* **9**, 329–340 (2013).

8. C. Peltz, C. Varin, T. Brabec, T. Fennel, Fully microscopic analysis of laser-driven finite plasmas using the example of clusters. *New J. Phys.* **14**, 065011 (2012).

9. A. H. Kelkar, L. Gulyas, L. C. Tribedi, Angle-differential observation of plasmon electrons in the double-differential cross-section spectra of fast-ion-induced electron ejection from C_{60}. *Phys. Rev. A* **92**, 052708 (2015).

10. S. Biswas, L. C. Tribedi, Plasmon-mediated electron emission from the coronene molecule under fast ion impact. *Phys. Rev. A* **92**, 060701(R) (2015).

11. S. Biswas, C. Champion, P. F. Weck, L. C. Tribedi, Differential electron emission from polycyclic aromatic hydrocarbon molecules under fast ion impact. *Sci. Rep.* **7**, 5560 (2017).

12. H. W. Kroto, J. R. Heath, S. C. O'brien, R. F. Curl, R. E. Smalley, C_{60}: Buckminsterfullerene. *Nature* **318**, 162–163 (1985).

13. J. Choi, E. H. Chang, D. M. Anstine, M. E.-A. Madjet, H. S. Chakraborty, Effects of exchange-correlation potentials on the density-functional description of C_{60} versus C_{240} photoionization. *Phys. Rev. A* **95**, 023404 (2017).

14. A. Ponzi, S. T. Manson, P. Decleva, Photoionization of C60: Effects of correlation on cross sections and angular distributions of valence subshells. *J. Phys. Chem. A* **124**, 108–125 (2020).

15. G. F. Bertsch, A. Bulgac, D. Tomanek, Y. Wang, Collective plasmon excitations in C_{60} clusters. *Phys. Rev. Lett.* **67**, 2690–2693 (1991).

16. I. V. Hertel, H. Steger, J. Devries, B. Weisser, C. Menzel, B. Kamke, W. Kamke, Giant plasmon excitation in free C_{60} and C_{70} molecules studied by photoionization. *Phys. Rev. Lett.* **68**, 784–787 (1992).

17. P. Lambin, A. A. Lucas, J. Vigneron, Polarization waves and van der Waals cohesion of C_{60} fullerite. *Phys. Rev. B* **46**, 1794–1803 (1992).

18. T. Liebsch, O. Plotzke, F. Heiser, U. Hergenhahn, O. Hemmers, R. Wehlitz, J. Viefhaus, B. Langer, S. B. Whitfield, U. Becker, Angle-resolved photoelectron-spectroscopy of C_{60}. *Phys. Rev. A* **52**, 457–464 (1995).

19. T. Barillot, C. Cauchy, P.-A. Hervieux, M. Gisselbrecht, S. E. Canton, P. Johnsson, J. Laksman, E. P. Mansson, J. M. Dahlstrom, M. Magrakvelidze, G. Dixit, M. E. Madjet, H. S. Chakraborty, E. Suraud, P. M. Dinh, P. Wopperer, K. Hansen, V. Loriot, C. Bordas, S. Sorensen, F. Lepine, Angular asymmetry and attosecond time delay from the giant plasmon resonance in C_{60} photoionization. *Phys. Rev. A* **91**, 033413 (2015).

20. M. Magrakvelidze, M. E. A. Madjet, G. Dixit, M. Ivanov, H. S. Chakraborty, Attosecond time delay in valence photoionization and photorecombination of argon: A time-dependent local-density-approximation study. *Phys. Rev. A* **91**, 063415 (2015).

21. E. P. Wigner, Lower limit for the energy derivative of the scattering phase shift. *Phys. Rev.* **98**, 145–147 (1955).

22. F. T. Smith, Lifetime matrix in collision theory. *Phys Rev* **118**, 349–356 (1960).

23. M. Schultze, M. Fiess, N. Karpowicz, J. Gagnon, M. Korbman, M. Hofstetter, S. Neppl, A. L. Cavalieri, Y. Komninos, T. Mercouris, C. A. Nicolaides, R. Pazourek, S. Nagele, J. Feist, J. Burgdörfer, A. M. Azzeer, R. Ernstorfer, R. Kienberger, U. Kleineberg, E. Goulielmakis, F. Krausz, V. S. Yakovlev, Delay in Photoemission. *Science* **328**, 1658–1662 (2010).

24. S. Biswas, B. Forg, L. Ortmann, J. Schotz, W. Schweinberger, T. Zimmermann, L. W. Pi, D. Baykusheva, H. A. Masood, I. Liontos, A. M. Kamal, N. G. Kling, A. F. Alharbi, M. Alharbi, A. M. Azzeer, G. Hartmann, H. J. Worner, A. S. Landsman, M. F. Kling, Probing molecular environment through photoemission delays. *Nat. Phys.* **16**, 778–783 (2020).

25. M. Hentschel, R. Kienberger, C. Spielmann, G. A. Reider, N. Milosevic, T. Brabec, P. Corkum, U. Heinzmann, M. Drescher, F. Krausz, Attosecond metrology. *Nature* **414**, 509–513 (2001).

26. R. Kienberger, E. Goulielmakis, M. Uiberacker, A. Baltuska, V. Yakovlev, F. Bammer, A. Scrinzi, T. Westerwalbesloh, U. Kleineberg, U. Heinzmann, M. Drescher, F. Krausz, Atomic transient recorder. *Nature* **427**, 817–821 (2004).

27. L. Seiffert, Q. Liu, S. Zherebtsov, A. Trabattoni, P. Rupp, M. C. Castrovilli, M. Galli, F. Sussmann, K. Wintersperger, J. Stierle, G. Sansone, L. Poletto, F. Frassetto, I. Halfpap, V. Mondes, C. Graf, E. Ruhl, F. Krausz, M. Nisoli, T. Fennel, F. Calegari, M. F. Kling, Attosecond chronoscopy of electron scattering in dielectric nanoparticles. *Nat. Phys.* **13**, 766–770 (2017).

28. M. Isinger, R. J. Squibb, D. Busto, S. Zhong, A. Harth, D. Kroon, S. Nandi, C. L. Arnold, M. Miranda, J. M. Dahlstrom, E. Lindroth, R. Feifel, M. Gisselbrecht, A. L'Huillier, Photoionization in the time and frequency domain. *Science* **358**, 893–896 (2017).

29. R. Pazourek, S. Nagele, J. Burgdörfer, Time-resolved photoemission on the attosecond scale: opportunities and challenges, in *Faraday Discussion 163* (Institut für Theoretische Physik, 2013), pp. 353–376.

30. G. Wachter, S. Nagele, S. A. Sato, R. Pazourek, M. Wais, C. Lemell, X. M. Tong, K. Yabana, J. Burgdorfer, Protocol for observing molecular dipole excitations by attosecond self-streaking. *Phys. Rev. A* **92**, 061403(R) (2015).

31. M. Huppert, I. Jordan, D. Baykusheva, A. von Conta, H. J. Wörner, Attosecond delays in molecular photoionization. *Phys. Rev. Lett.* **117**, 093001 (2016).

32. I. J. Sola, E. Mevel, L. Elouga, E. Constant, V. Strelkov, L. Poletto, P. Villoresi, E. Benedetti, J. P. Caumes, S. Stagira, C. Vozzi, G. Sansone, M. Nisoli, Controlling attosecond electron dynamics by phase-stabilized polarization gating. *Nat. Phys.* **2**, 319–322 (2006).

33. G. Sansone, E. Benedetti, F. Calegari, C. Vozzi, L. Avaldi, R. Flammini, L. Poletto, P. Villoresi, C. Altucci, R. Velotta, S. Stagira, S. De Silvestri, M. Nisoli, Isolated single-cycle attosecond pulses. *Science* **314**, 443–446 (2006).

34. J. Piella, N. G. Bastus, V. Puntes, Size-controlled synthesis of sub-10-nanometer citrate-stabilized gold nanoparticles and related optical properties. *Chem. Mater.* **28**, 1066–1075 (2016).

35. R. Shaik, H. R. Varma, H. S. Chakraborty, Comparative study of plasmon-resonance properties as a function of fullerene size using density functional theory (APS Division of Atomic, Molecular and Optical Physics Meeting, 2022).

36. M. Lucchini, M. H. Brügmann, A. Ludwig, L. Gallmann, U. Keller, T. Feurer, Ptychographic reconstruction of attosecond pulses. *Opt. Express* **23**, 29502–29513 (2015).

37. J. Schötz, B. Förg, W. Schweinberger, I. Liontos, H. A. Masood, A. M. Kamal, C. Jakubeit, N. G. Kling, T. Paasch-Colberg, S. Biswas, M. Högner, I. Pupeza, M. Alharbi, A. M. Azzeer, M. F. Kling, Phase-matching for generation of isolated attosecond XUV and soft-x-ray pulses with few-cycle drivers. *Phys. Rev. X* **10**, 041011 (2020).

38. G. Wachter, Simulation of condensed matter dynamics in strong femtosecond laser pulses, thesis, Vienna University of Technology, Austria (2014).

39. M. Magrakvelidze, D. M. Anstine, G. Dixit, M. E. Madjet, H. S. Chakraborty, Attosecond structures from the molecular cavity in fullerene photoemission time delay. *Phys. Rev. A* **91**, 053407 (2015).

40. M. F. Ciappina, A. Becker, A. Jaron-Becker, Multislit interference patterns in high-order harmonic generation in C_{60}. *Phys. Rev. A* **76**, 063406 (2007).

41. M. F. Ciappina, A. Becker, A. Jaron-Becker, High-order harmonic generation in fullerenes with icosahedral symmetry. *Phys. Rev. A* **78**, 063405 (2008).

42. O. Gunnarsson, B. I. Lundqvist, Exchange and correlation in atoms, molecules, and solids by spin-density functional formalism. *Phys Rev B* **13**, 4274–4298 (1976).

43. R. Vanleeuwen, E. J. Baerends, Exchange-correlation potential with correct asymptotic-behavior. *Phys. Rev. A* **49**, 2421–2431 (1994).

44. G. L. Oliver, J. P. Perdew, Spin-density gradient expansion for the kinetic-energy. *Phys. Rev. A* **20**, 397–403 (1979).

45. M. A. L. Marques, A. Castro, A. Rubio, Assessment of exchange-correlation functionals for the calculation of dynamical properties of small clusters in time-dependent density functional theory. *J. Chem. Phys.* **115**, 3006–3014 (2001).

46. N. Troullier, J. L. Martins, Structural and electronic-properties of C_{60}. *Phys Rev B* **46**, 1754–1765 (1992).

47. J. H. Weaver, J. L. Martins, T. Komeda, Y. Chen, T. R. Ohno, G. H. Kroll, N. Troullier, R. E. Haufler, R. E. Smalley, Electronic-structure of solid C_{60}—Experiment and theory. *Phys. Rev. Lett.* **66**, 1741–1744 (1991).

48. M. Vos, S. A. Canney, I. E. McCarthy, S. Utteridge, M. T. Michalewicz, E. Weigold, Electron-momentum spectroscopy of fullerene. *Phys Rev B* **56**, 1309–1315 (1997).

49. A. Zangwill, P. Soven, Density-functional approach to local-field effects in finite systems—Photoabsorption in the rare-gases. *Phys. Rev. A* **21**, 1561–1572 (1980).

50. W. Ekardt, Size-dependent photoabsorption and photoemission of small metal particles. *Phys Rev B* **31**, 6360–6370 (1985).

51. T. Nakatsukasa, K. Yabana, Photoabsorption spectra in the continuum of molecules and atomic clusters. *J. Chem. Phys.* **114**, 2550–2561 (2001).

52. M. E. Madjet, H. S. Chakraborty, J. M. Rost, S. T. Manson, Photoionization of C_{60}: A model study. *J. Phys. B At. Mol. Opt. Phys.* **41**, 105101 (2008).

53. A. Rudel, R. Hentges, U. Becker, H. S. Chakraborty, M. E. Madjet, J. M. Rost, Imaging delocalized electron clouds: Photoionization of C_{60} in Fourier reciprocal space. *Phys. Rev. Lett.* **89**, 125503 (2002).

54. S. Korica, A. Reinkoester, M. Braune, J. Viefhaus, D. Rolles, B. Langer, G. Fronzoni, D. Toffoli, M. Stener, P. Decleva, O. M. Al-Dossary, U. Becker, Partial photoionization cross sections of C_{60} and C_{70}: A gas versus adsorbed phase comparison. *Surf. Sci.* **604**, 1940–1944 (2010).

55. S. W. J. Scully, E. D. Emmons, M. F. Gharaibeh, R. A. Phaneuf, A. L. D. Kilcoyne, A. S. Schlachter, S. Schippers, A. Muller, H. S. Chakraborty, M. E. Madjet, J. M. Rost, Photoexcitation of a volume plasmon in C_{60} ions. *Phys. Rev. Lett.* **94**, 065503 (2005).

56. G. Wendin, Collective effects in atomic photoabsorption spectra .3. Collective resonance in 4 D-10 Shell in Xe. *J. Phys. B At. Mol. Opt. Phys.* **6**, 42–61 (1973).

57. J. Watzel, A. S. Moskalenko, Y. Pavlyukh, J. Berakdar, Angular resolved time delay in photoemission. *J. Phys. B At. Mol. Opt. Phys.* **48**, 025602 (2015).

58. U. Fano, Effects of Configuration Interaction on Intensities and Phase Shifts. *Phys Rev.* **124**, 1866–1878 (1961).

59. J. U. Andersen, E. Bonderup, Classical dielectric models of fullerenes and estimation of heat radiation. *Eur. Phys. J. D At. Mol. Opt. Phys.* **11**, 413–434 (2000).

60. E. Maurat, P. A. Hervieux, F. Lepine, Surface plasmon resonance in C_{60} revealed by photoelectron imaging spectroscopy. *J. Phys. B At. Mol. Opt. Phys.* **42**, 165105 (2009).

61. P. Drude, On the electron theory of metals. *Ann. Phys-Berlin.* **306**, 566–613 (1900).

62. A. Ballard, K. Bonin, J. Louderback, Absolute measurement of the optical polarizability of C_{60}. *J. Chem. Phys.* **113**, 5732–5735 (2000).

63. J. R. Cash, A. H. Karp, R. J. Renka, A Variable order Runge-Kutta method for initial-value problems with rapidly varying right-hand sides. *ACM Trans. Math Softw.* **16**, 201–222 (1990).

64. C. Runge, Ueber die numerische Auflösung von Differentialgleichungen. *Math. Ann.* **46**, 167–178 (1895).

65. W. Kutta, Beitrag zur näherungweisen Integration totaler Differentialgleichungen.t*Z. Angew. Math. Phys.* **46**, 435–453 (1901).

66. M. A. L. Marques, N. T. Maitra, F. Nogueira, E. K. U. Gross, A. Rubio, Fundamentals of time-dependent density functional theory (Springer-Verlag, 2012).

67. P. Wopperer, U. De Giovannini, A. Rubio, Efficient and accurate modeling of electron photoemission in nanostructures with TDDFT. *Eur. Phys. J. B.* **90**, 1307 (2017).

68. L. Tao, A. Scrinzi, Photo-electron momentum spectra from minimal volumes: The time-dependent surface flux method. *New J. Phys.* **14**, 013021 (2012).

69. N. Tancogne-Dejean, M. J. T. Oliveira, X. Andrade, H. Appel, C. H. Borca, G. Le Breton, F. Buchholz, A. Castro, S. Corni, A. A. Correa, U. De Giovannini, A. Delgado, F. G. Bich, J. Flick, G. Gil, A. Gomez, N. Helbig, H. Hubener, R. Jestadt, J. Jornet-Somoza, A. H. Larsen, I. V. Lebedeva, M. Luders, M. A. L. Marques, S. T. Ohlmann, S. Pipolo, M. Rampp, C. A. Rozzi, D. A. Strubbe, S. A. Sato, C. Schafer, I. Theophilou, A. Welden, A. Rubio, Octopus, a computational framework for exploring light-driven phenomena and quantum dynamics in extended and finite systems. *J. Chem. Phys.* **152**, 124119 (2020).

70. A. Crawford-Uranga, U. De Giovannini, E. Rasanen, M. J. T. Oliveira, D. J. Mowbray, G. M. Nikolopoulos, E. T. Karamatskos, D. Markellos, P. Lambropoulos, S. Kurth, A. Rubio, Time-dependent density-functional theory of strong-field ionization of atoms by soft x rays. *Phys. Rev. A* **90**, 033412 (2014).

71. U. De Giovannini, A. H. Larsen, A. Rubio, Modeling electron dynamics coupled to continuum states in finite volumes with absorbing boundaries. *Eur. Phys. J. B.* **88**, 56 (2015).

72. A. Reinkoster, S. Korica, G. Prumper, J. Viefhaus, K. Godehusen, O. Schwarzkopf, M. Mast, U. Becker, The photoionization and fragmentation of C_{60} in the energy range 26-130 eV.*J. Phys. B At. Mol. Opt. Phys.* **37**, 2135–2144 (2004).
